# In Situ Vaccination with a Vpr-Derived Peptide Elicits Systemic Antitumor Immunity by Improving Tumor Immunogenicity

**DOI:** 10.3390/vaccines13070710

**Published:** 2025-06-30

**Authors:** Danjie Pan, Ling Du, Jiayang Liu, Kudelaidi Kuerban, Xuan Huang, Yue Wang, Qiuyu Guo, Huaning Chen, Songna Wang, Li Wang, Pinghong Zhou, Zhefeng Meng, Li Ye

**Affiliations:** 1 Department of Biological Medicines, School of Pharmacy, Minhang Branch, Zhongshan Hospital, Fudan University, Shanghai 201199, China; 21211030089@m.fudan.edu.cn (D.P.); 22111510062@m.fudan.edu.cn (J.L.); kudelaidi@fudan.edu.cn (K.K.); 19211030087@fudan.edu.cn (X.H.); 21211030099@m.fudan.edu.cn (Y.W.); 21211030071@m.fudan.edu.cn (Q.G.); 21211030059@m.fudan.edu.cn (H.C.); 2 School of Pharmacy and Laboratory of Drug Discovery from Natural Resources and Industrialization, Macau University of Science and Technology, Macau SAR 999078, China; 3230006875@student.must.edu.mo; 3 Endoscopy Center and Endoscopy Research Institute, Zhongshan Hospital, Fudan University, Shanghai 200032, China; 21111210004@m.fudan.edu.cn (L.D.); 21111210167@m.fudan.edu.cn (L.W.); zhou.pinghong@zs-hospital.sh.cn (P.Z.); 4 Endoscopy Center, Shanghai Geriatric Medical Center, Shanghai 201104, China; 5 Shanghai Collaborative Innovation Center of Endoscopy, Shanghai 200032, China

**Keywords:** viral protein R, cell apoptosis, immunogenic cell death, tumor immunotherapy, tumor vaccine

## Abstract

**Background:** Cancer vaccines represent a groundbreaking advancement in cancer immunotherapy, utilizing tumor antigens to induce tumor-specific immune responses. However, challenges like tumor-induced immune resistance and technical barriers limit the widespread application of predefined antigen vaccines. Here, we investigated the potential of viral protein R (Vpr) peptides as effective candidates for constructing anonymous antigen vaccines in situ by directly injecting at the tumor site and releasing whole-tumor antigens, inducing robust anti-tumor immune responses to overcome the limitations of predefined antigen vaccines. **Methods:** The cytotoxic effects of Vpr peptides were evaluated using the CCK8 reagent kit. Membrane penetration ability of Vpr peptides was observed using a confocal laser scanning microscope and quantitatively analyzed using flow cytometry. EGFR levels in the cell culture supernatants of cells treated with Vpr peptides were evaluated using an ELISA. Surface exposure of CRT on the tumor cell surface was observed using a confocal laser scanning microscope and quantitatively analyzed using flow cytometry. The secretion levels of ATP from tumor cells were evaluated using an ATP assay kit. HMGB1 release was evaluated using an ELISA. Mouse (Male C57BL/6 mice aged 4 weeks) MC38 and LLC bilateral subcutaneous tumor models were established to evaluate the therapeutic effects of Vpr peptides through in situ vaccination. Proteomic analysis was performed to explore the mechanism of anti-tumor activity of Vpr peptides. **Results:** Four Vpr peptides were designed and synthesized, with P1 and P4 exhibiting cytotoxic effects on tumor cells, inducing apoptosis and immunogenic cell death. In mouse tumor models, in situ vaccination with Vpr peptide significantly inhibited tumor growth and activated various immune cells. High-dose P1 monotherapy demonstrated potent anti-tumor effects, activating DCs, T cells, and macrophages. Combining ISV of P1 with a CD47 inhibitor SIRPαFc fusion protein showed potent distant tumor suppression effects. Proteomic analysis suggested that Vpr peptides exerted anti-tumor effects by disrupting tumor cell morphology, movement, and adhesion, and promoting immune cell infiltration. **Conclusions:** The designed Vpr peptides show promise as candidates for in situ vaccination, with significant anti-tumor effects, immune activation, and favorable safety profiles observed in mouse models. In situ vaccination with Vpr-derived peptides represents a potential approach for cancer immunotherapy.

## 1. Introduction

In recent years, global cancer incidence has been severe, with both incidence and mortality rates showing a continuous upward trend, posing a major threat to human health [1]. As a research hotspot over the past two decades, tumor immunotherapy, including immune checkpoint inhibitors, adoptive cell therapy, and cancer vaccines, has become an effective clinical strategy for cancer treatment [2,3]. Among these, cancer vaccines represent a significant advancement in cancer immunotherapy. Therapeutic cancer vaccines induce the regression of existing malignant tumors by activating anti-tumor immune responses [4]. Benefiting from advancements in vaccine platforms and antigen screening technologies, the efficacy of therapeutic cancer vaccines has also been enhanced [5,6]. Combining therapeutic vaccines with other immunotherapies such as immune checkpoint blockade therapy has shown promising results in enhancing anti-tumor responses [4]. Based on our knowledge of tumor-specific antigens, therapeutic cancer vaccines can be classified into predefined (known) antigen vaccines and anonymous (unknown) antigen vaccines [7].

Cancer vaccines aim to induce tumor regression while establishing durable anti-tumor immune memory to avoid nonspecific and adverse reactions [8]. However, as critically analyzed in our previous review [9], tumor-induced immune resistance and suppression limit the effectiveness of predefined antigen vaccines. The development of predefined vaccines faces various technical challenges such as the high cost of sequencing technologies and epitope prediction technologies for cancer vaccine production and the time-consuming process of preparing tumor lysates [10,11,12,13,14]. Additionally, insufficient understanding of the tumor antigen epitopes that constitute immunogenicity and uncertainty about whether new antigens can be effectively presented by MHC pose challenges [15,16]. Therefore, addressing the shortcomings of existing cancer vaccines and exploring technologies for more effective and safer cancer vaccines is a pressing issue in current cancer vaccine development.

In this context, anonymous antigen vaccines in situ hold great promise [9]. Anonymous antigen cancer vaccines in situ aim to activate anti-tumor immune responses by initiating or stimulating immune responses at the tumor site using a wide range of potential tumor-associated antigens (TAAs) or tumor-specific antigens (TSAs) through a method known as in situ vaccination (ISV) [17]. Anonymous antigen cancer vaccines do not limit themselves to individual TAAs or TSAs but utilize the antigen repertoire of each patient’s tumor to induce specific anti-tumor immune responses with patient specificity [7,18]. To trigger robust memory anti-tumor immune responses, in situ vaccines often induce immunogenic cell death (ICD) in tumors to promote the release of TAAs and enhance antigen-presenting cell uptake and activation of antigens, thereby inducing T cell responses and activating systemic anti-tumor immunity [19]. ICD refers to the process where non-immunogenic cell death becomes immunogenic due to external stimuli leading to the release of damage-associated molecular patterns (DAMPs) such as calreticulin (CRT), ATP secretion, and high mobility group protein B1 (HMGB1) [20,21,22,23,24,25]. DAMPs bind to pattern recognition receptors on dendritic cells (DCs), generating immune activation signals that ultimately activate innate and adaptive immune responses [26,27].

We utilized viral protein R (Vpr) to construct an anonymous antigen vaccine in situ. Vpr is an essential regulatory protein of HIV-1 with functions like cell membrane penetration, cell cycle regulation, and induction of apoptosis that align with the requirements for constructing an anonymous vaccine in situ [28,29,30,31,32]. The molecular structure of Vpr includes three alpha helices, a flexible N-terminal region, and a C-terminal basic amino acid region [33,34]. Its domains for apoptosis induction, nuclear localization signal, and transmembrane domain are found in different structural regions where Vpr interacts with intracellular transcription factors like NF-κB or apoptotic factors like Bax to regulate cellular states and exhibit immunomodulatory functions [35,36,37,38]. Studies on potential anti-tumor activity of Vpr in vitro suggest that Vpr selectively inhibits the growth of rapidly dividing tumor cells without affecting normal cells, while the mechanism still needs further study [39,40,41]. This characteristic gives Vpr significant potential as an anti-tumor drug due to its specificity towards anti-apoptotic factors highly expressed in proliferating cells [42,43]. Moreover, the apoptosis-inducing effect of Vpr is independent of specific intracellular factors, making it less prone to apoptosis resistance mechanisms [42,44]. Additionally, the inherent ability of Vpr for cell membrane transduction allows it to enter tumor cells directly without external carriers facilitating its therapeutic action [30,42,45]. Numerous studies have confirmed the ability of Vpr to induce apoptosis in tumor cells while penetrating cell membranes to exert its anti-tumor effects [30,39,40,46,47]. Therefore, Vpr protein represents a promising candidate for cancer immunotherapy.

Given this background, Vpr, especially its transmembrane and apoptosis-inducing domains, may be ideal for constructing novel anonymous antigen vaccines. Herein, we screened and constructed innovative Vpr peptides with transmembrane domains and apoptosis-inducing domains to validate their potential application as anti-tumor peptide drugs by penetrating tumor cell membranes and inducing apoptosis in tumor cells. Furthermore, we investigated the function of Vpr peptide in promoting the release of tumor cell antigens and inducing ICD in tumor cells to enhance their immunogenicity, aiming to test the feasibility of constructing anonymous antigen vaccines in situ based on Vpr peptides. Subsequently, we used the Vpr peptides for ISV in various transplanted tumor models and attempted to combine them with immune checkpoint inhibitors through combination therapy to examine the immunotherapeutic effect, providing a new design for cancer vaccine research and a new approach to cancer immunotherapy.

## 2. Materials and Methods

### 2.1. Viral Protein R

The peptides were commercially synthesized (Nanjing, China), enriched to >90% purity, and delivered as lyophilized trifluoroacetate salt (powder) [31]. For the CLSM experiments, the peptides were labeled with FITC when synthesized. All peptides were fully dissolved at working concentrations (≤40 μM in vitro, ≤15 mg/kg in vivo). Solutions were prepared in cell culture medium DMEM or PBS. The sequences of peptides used in this study are shown in Table 1.

### 2.2. Cell Lines and Treatment

HT-29 (human colorectal adenocarcinoma cells), MC38 (murine colon carcinoma cells), B16F10 (murine melanoma cells) and LLC (Lewis Lung Carcinoma) cell lines were purchased from the Cell Bank of the Chinese Academy of Sciences (Shanghai, China). DMEM medium (Corning, Corning, NY, USA) supplemented with 100 IU/mL penicillin (Beyotime, Shanghai, China) and 10% fetal bovine serum (Gibco, Grand Island, NY, USA) was used. The cells were incubated at 37 °C with 5% CO_2_ in a cell incubator.

### 2.3. Cytotoxicity Assay

The cytotoxicity of Vpr peptides against HT-29 cells was measured using the CCK8 reagent (Beyotime, Shanghai, China). HT-29 cells (5 × 10^4^/mL) were added to a 96-well plate with 100 μL of cell suspension per well. After treating the cells with the peptides at different concentrations (0.1–100 μM) for 72 h, CCK-8 reagent was added, with 10 μL for each pore. Then, the mixture was incubated in an incubator at 37 °C for 1.5 h, and the absorbance at 450 nm was measured using a microplate reader.

### 2.4. Apoptosis Assay

After 72 h of treatment with 50 μM of Vpr peptides, the cells (HT-29, MC38, CT-26) were digested using 0.25% trypsin and collected via centrifugation at 1500 rpm. Subsequently, they were washed twice with PBS and stained with Annexin V-FITC and PI for 20 min at 4 °C in the dark. Flow cytometry (Becton-Dickinson, Franklin Lakes, NJ, USA) was used to evaluate the level of apoptosis.

### 2.5. Transmembrane Assay

HT-29 cells (5 × 10^4^/mL) were added to a confocal plate with 100 μL of cell suspension per well. After treating the cells with the peptides at 40 μM for 2 h, the cells were washed twice with PBS and stained with Hoechst 33342 for 15 min to provide nuclear counterstaining for cellular localization reference in confocal imaging and observe nuclear condensation/fragmentation. Afterward, the cells were washed with PBS and imaged using a confocal laser scanning microscope (Olympus, Tokyo, Japan).

A total of 1 × 10^6^ HT-29 cells were collected in 1.5 mL microcentrifuge tubes. The cells were resuspended in 200 μL of 40 μM FITC-labeled Vpr peptide suspension. Following incubation at 37 °C for 2 h, the peptides were washed off. Flow cytometry analysis was conducted using a Beckman CytoFlex S Flow Cytometer (Brea, CA, USA), and the data were analyzed using CytExpert Software (2.4.0.28).

### 2.6. EGFR Release Evaluation

Cancer cells (HT-29) were treated with Vpr peptides at concentrations of 10, 20, and 40 μM for 48 h. EGFR levels in the cell culture supernatants were subsequently quantified using an EGFR ELISA detection kit (MultiSciences, EK1192-96, Hangzhou, China) according to the manufacturer’s instructions.

### 2.7. CRT Exposure Assay

Cancer cells (MC38, B16F10, LLC) were treated with 40 μM of various Vpr peptides for 48 h, and the cells were washed with PBS and fixed with 4% paraformaldehyde for 15 min at room temperature. Subsequently, the cells were permeabilized with 0.1% Triton X-100 in PBS for 10 min and then incubated with blocking buffer containing 5% bovine serum albumin (BSA) for 1 h at room temperature. The cell samples were then incubated overnight at 4 °C with an anti-calreticulin antibody (Beyotime, AF1666, Shanghai, China). Afterward, the cells were washed three times with PBS and incubated with secondary antibody (Abmart, M21014M, Shanghai, China) for 1 h at room temperature in the dark. Finally, the cells were stained with DAPI, washed with PBS, and imaged using a confocal laser scanning microscope (Olympus, Tokyo, Japan).

Cancer cells (MC38, B16F10, LLC) were treated with 40 μM of various Vpr peptide sequences for 48 h. Subsequently, the cells were incubated with anti-calreticulin antibody (Beyotime, AF1666, Shanghai, China) at room temperature for 1 h. After washing three times with PBS, the cells were incubated with secondary antibody (Abmart, M21014M, Shanghai, China) for 1 h at room temperature in the dark and washed with PBS. Flow cytometry analysis was conducted using the Beckman CytoFlex S Flow Cytometer, and the data were analyzed using CytExpert Software.

### 2.8. ATP and HMGB1 Evaluation

Cancer cells (MC38, B16F10, LLC) were treated with 40 μM Vpr peptides for 48 h. ATP levels in the cell culture supernatants were subsequently quantified using an ATP detection kit (cominbio, ATP-1-Y, Suzhou, China) according to the manufacturer’s instructions. HMGB1 levels in the cell culture supernatants were subsequently quantified using an HMGB1 detection kit (lengton, BPE20487, Shanghai, China) according to the manufacturer’s instructions.

### 2.9. Phagocytosis Test

1 × 10^6^ LLC cells were resuspended in 1 mL of cell labeling solution. Separately, 2 μL of CFDA-SE was added to 1 mL of cell labeling solution, mixed thoroughly, and then added to the cell suspension. The mixture was incubated at 37 °C for 5 min. Subsequently, 5 mL of complete DMEM was added to wash the cells three times to complete the CFDA-SE labeling of LLC tumor cells. The labeled tumor cells were then treated with 40 μM of Vpr peptides P1 and P4 for 24 h. Ana-1 macrophages (1 × 10^5^ cells) were pre-seeded into confocal dishes and allowed to adhere for 8 to 12 h. Following adherence, the macrophages were subjected to a 2 h serum starvation period in serum-free medium. Post-starvation, 2 × 10^5^ CFDA SE-labeled LLC cells were introduced into the culture, and co-incubation was carried out in complete medium, either without any treatment (control group) or with 40 μM Vpr peptides for a duration of 2 h. Subsequently, the dishes were washed with PBS to remove non-phagocytosed tumor cells. The samples were then examined using CLSM. The number of macrophages that had phagocytosed tumor cells was quantified, and a phagocytic index was calculated, defined as the number of macrophages that had engulfed tumor cells per 100 macrophages.

### 2.10. In Vivo Experiments

In the bilateral flank MC38 tumor model and LLC tumor model, tumors were established by injecting 2 × 10^6^ cells (MC38 or LLC) subcutaneously into the right flank and 1 × 10^6^ cells into the left flank. C57BL/6 mice (five weeks old, male) were randomly assigned to different treatment groups, and treatment began at a mean tumor burden of 100 mm^3^ twice per week for 2 weeks. Five mice per group (n = 5) were used for all in vivo experiments. Tumor size was measured using digital calipers, and volumes (mm^3^) were calculated according to the following formula: V = 0.5 × length (L) × width (W)^2^. The mice’s weight loss was assessed twice a week. Baseline body weight was recorded before tumor cell administration. Animals exhibiting clinical signs of xGVHD (≥20% weight loss, hunched posture, reduced mobility, fur loss, tachypnea) were euthanized via CO_2_ inhalation. Administration to the mice was performed as described below.
**Model****Group****Administration**MC38 tumor modelControl50 μL 1× PBS (right flank, i.t.)Poly(I:C)2.5 mg/kg Poly(I:C) (s.c.)P1 + Poly(I:C)10 mg/kg P1 (right flank, i.t.) +2.5 mg/kg Poly(I:C) (s.c.)P4 + Poly(I:C)15 mg/kg P4 (right flank, i.t.) +2.5 mg/kg Poly(I:C) (s.c.)LLC tumor modelControl50 μL 1× PBS (right flank, i.t.)P1-1010 mg/kg P1 (right flank, i.t.)P1-1515 mg/kg P1 (right flank, i.t.)P1 + Poly(I:C)10 mg/kg P1 (right flank, i.t.) +2.5 mg/kg Poly(I:C) (s.c.)LLC tumor modelControl50 μL 1× PBS (right flank, i.t.)P110 mg/kg P1 (right flank, i.t.)SIRPαFc10 mg/kg SIRPαFc (i.p.)P1 + SIRPαFc10 mg/kg P1 (right flank i.t.) +10 mg/kg SIRPαFc (i.p.)

### 2.11. Flow Cytometry

To prepare single-cell suspensions, fresh tumors, lymph nodes, and spleens were ground and filtered through a 70 μm cell strainer (Falcon, Catalog: 352350, Corning, NY, USA). Antibodies against the following molecules were used according to the manufacturer’s instructions: These included anti-mouse CD45 (violetFluor 450-labeled, MultiSciences, Catalog: F2104507, Shanghai, China), anti-mouse CD3 (FITC-labeled, MultiSciences, Catalog: F2100301, Shanghai, China), anti-mouse CD4 (PerCP-Cy5.5-labeled, MultiSciences, Catalog: F2100404, Shanghai, China), anti-mouse CD4 (APC-Cy7-labeled, MultiSciences, Catalog: F2100406, Shanghai, China), anti-mouse CD8 (PE-Cy7-labeled, MultiSciences, Catalog: F2100805, Shanghai, China), anti-mouse CD8 (PE-Cy5-labeled, MultiSciences, Catalog: F2100810, Shanghai, China), anti-mouse CD69 (PE-labeled, MultiSciences, Catalog: F2106902, Shanghai, China), anti-mouse CD11c (APC-labeled, MultiSciences, Catalog: F21011C03, Shanghai, China), anti-mouse CD103 (PE-labeled, Biolegend, Catalog: 121406, San Diego, CA, USA), anti-human/mouse CD11b (PerCP-Cy5.5-labeled, MultiSciences, Catalog: F41011B04, Shanghai, China), anti-human/mouse CD11b (PE-Cy7-labeled, MultiSciences, Catalog: F41011B05, Shanghai, China), anti-mouse F4/80 (PE-Cy7-labeled, MultiSciences, Catalog: F21480A05, Shanghai, China), anti-mouse H-2Kb/H-2Db (PerCP/Cyanine5.5-labeled, Biolegend, Catalog: 114620, San Diego, CA, USA), and anti-mouse MHC II (FITC-labeled, MultiSciences, Catalog: F21IIAE01, Shanghai, China). Flow cytometry analysis was conducted using a Beckman CytoFlex S Flow Cytometer, and the data were analyzed using CytExpert Software.

### 2.12. IHC Analysis

Paraffin-embedded tumor and spleen sections of 5 μm thickness were prepared. IHC was performed to evaluate the expression of CD4, CD8, F4/80, CD11c, Ki67, CD47, CRT, and HMGB1. Images were captured using an Inverted Phase Contrast Fluorescence Microscope (Olympus, Tokyo, Japan). The positive area proportions were quantified using ImageJ software (v2022.06.01) and analyzed via one-way ANOVA using GraphPad Prism 9 software.

### 2.13. H&E Staining Analysis

Paraffin-embedded major organ specimens, including the heart, liver, spleen, lung, and kidney, were sectioned at a 5 μm thickness, then Hematoxylin and Eosin (H&E) staining was performed for a necrosis study to evaluate drug-related damage. Images of stained slices were acquired using an inverted phase contrast fluorescence microscope (Olympus).

### 2.14. Proteome Analysis

The tumor samples collected from the LLC-transplanted tumor model efficacy study in mice were subjected to proteomic analysis by Jingjie Biotechnology Company (Hangzhou, China). The proteins identified in the samples through trypsin digestion and liquid chromatography-mass spectrometry were analyzed via database searching and bioinformatics analysis. The ratio values of P1/control were calculated, with fold-change > 1.5 or fold-change < 0.67 and *p* < 0.05 considered significant changes. These differentially phosphorylated proteins underwent GO and KEGG enrichment analysis (fold change = Log2 ratio).

### 2.15. Statistical Analysis

The data in this study are presented as means ± standard deviations (SD). Statistical significance between groups was determined using GraphPad Prism 9 (GraphPad Software Inc., La Jolla, CA, USA). A *p* value < 0.05 was considered statistically significant.

## 3. Results

### 3.1. Vpr Peptides Induced Apoptosis of Tumor Cells

Firstly, we designed and synthesized four Vpr peptides, named P1, P2, P3, and P4. The peptide P1 contains structural domains that have been previously reported to be potentially related to the transmembrane and apoptosis-inducing functions of the Vpr protein [31,48,49]. The peptides P2 and P3 are associated with transmembrane and apoptosis-inducing functions, respectively [40,46,50]. The peptide P4 is based on the P1 peptide. Still, it includes an additional segment from the N-terminus of the Vpr protein that may be related to apoptosis induction [51]. The peptides were designed according to our previous study by Ling Du [51]. The amino acid sequences for the peptides tested are presented in Table 1. To improve the solubility of the peptides, all four peptides were prepared as trifluoroacetic acid salts.

**Figure 1:**
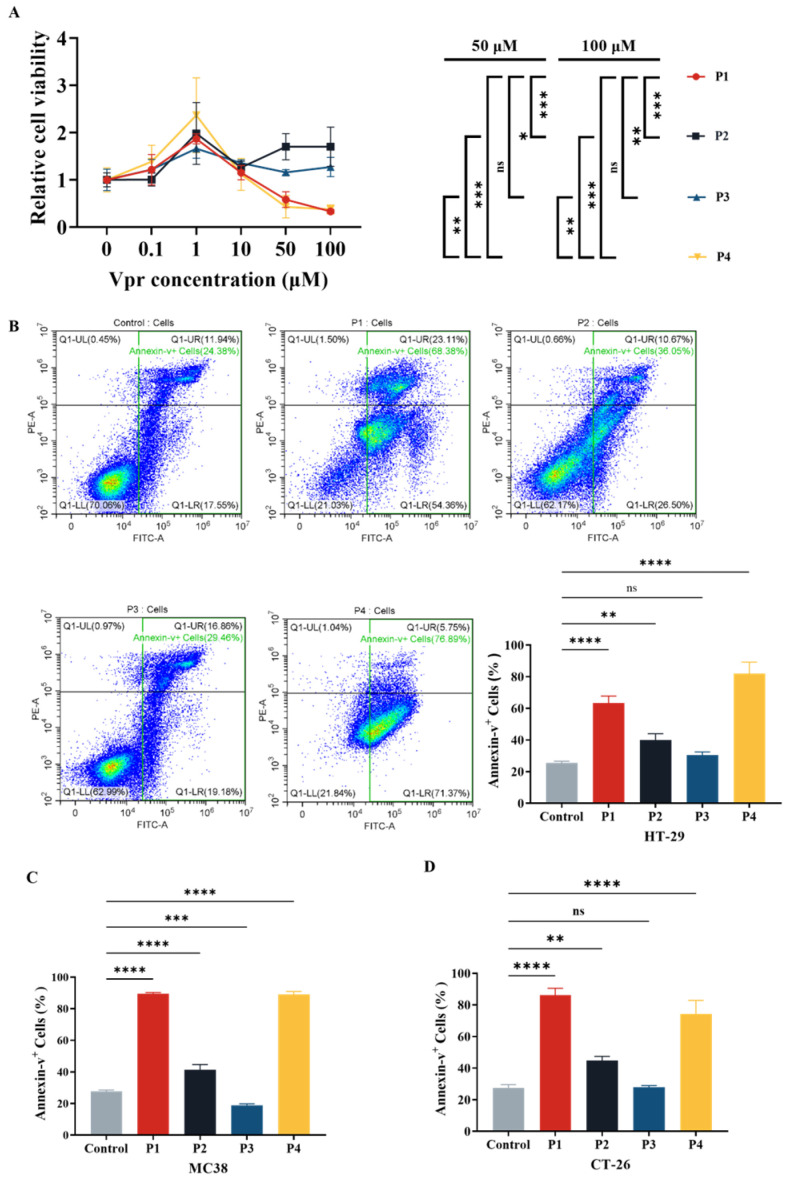
Vpr peptide-induced apoptosis of tumor cells. (**A**) After treatment with Vpr peptides in different concentrations for 72 h, the cell viability of HT29 cells was evaluated based on the CCK8 assay (n = 3). (**B**–**D**) After treatment with 40 μM Vpr peptides for 72 h, HT-29 (**B**), MC38 (**C**), and CT-26 (**D**) cells were stained with Annexin V/PI, and apoptosis was analyzed using flow cytometry (n = 3). The data are presented as means ± SD, ns: no significance, * *p* < 0.0332, ** *p* < 0.0021, *** *p* < 0.0002, **** *p* < 0.0001.

After the four Vpr peptides were synthesized, their cytotoxic effects were evaluated using the CCK8 reagent kit. At concentrations ranging from 0 to 1 μM, all four Vpr peptides promoted proliferation of human colorectal cancer HT-29 cells (Figure 1A). At concentrations above 1 μM, P1 and P4 showed inhibitory effects on HT-29 cell proliferation, displaying a certain concentration dependency, while P2 and P3 did not exhibit significant inhibitory effects on cell proliferation (Figure 1A). The characteristics displayed by P1 and P4 are consistent with previous studies on full-length Vpr protein: low doses of Vpr have anti-apoptotic effects, while high doses induce cell apoptosis [49,52]. Based on one-way ANOVA, at concentrations of 50 μM and 100 μM, the cell proliferation levels in the P1 and P4 groups were significantly lower than those in the P2 and P3 groups, with no significant difference between the P1 and P4 groups (Figure 1A). This result indicates that at high concentrations (1–100 μM), the Vpr peptides P1 and P4 exhibit concentration-dependent cytotoxic effects, significantly inhibiting the proliferation of colon cancer cells HT29, while P2 and P3 do not show this effect.

Based on the results of the cytotoxicity experiment (Figure 1A), we evaluated the effects of Vpr peptides on inducing apoptosis in tumor cells at a concentration of 40 μM using flow cytometry. The results showed that compared to the control group, treatment with 40 μM P1 increased the total apoptosis rate of HT-29 cells from 25.37% to 63.32%, while P4 treatment raised it to 81.97%. Both P1 and P4 significantly promoted apoptosis in HT-29 cells. Treatment with P2 resulted in a total apoptosis rate of 39.93%, and P3 treatment led to 30.44% (Figure 1B). Similarly, we validated the effect of Vpr peptide-induced tumor cell apoptosis on two other mouse colon cancer cell lines, MC38 and CT26, using flow cytometry. The results indicated that after treatment with 40 μM P1, the total apoptosis rate of MC38 cells increased from 27.66% to 89.32%, reaching 88.99% with P4 treatment. Both P1 and P4 exhibited significant pro-apoptotic effects on MC38 cells. Treatment with P2 resulted in a total apoptosis rate of 41.37% for MC38, while P3 treatment led to 18.89% (Figure 1C). For CT26 cells, after treatment with 40 μM P1, the total apoptosis rate increased from 27.54% to 86.20%, reaching 74.36% with P4 treatment. Both P1 and P4 showed significant pro-apoptotic effects on CT26 cells. Treatment with P2 resulted in a total apoptosis rate of 44.78% for CT26, while P3 treatment led to 27.81% (Figure 1D). These results demonstrate that Vpr peptides P1 and P4 can induce apoptosis in various colon cancer cells, while P2 and P3 did not exhibit significant pro-apoptotic effects.

### 3.2. Vpr Peptides Penetrated the Tumor Cell Membrane and Entered the Cells

**Figure 2:**
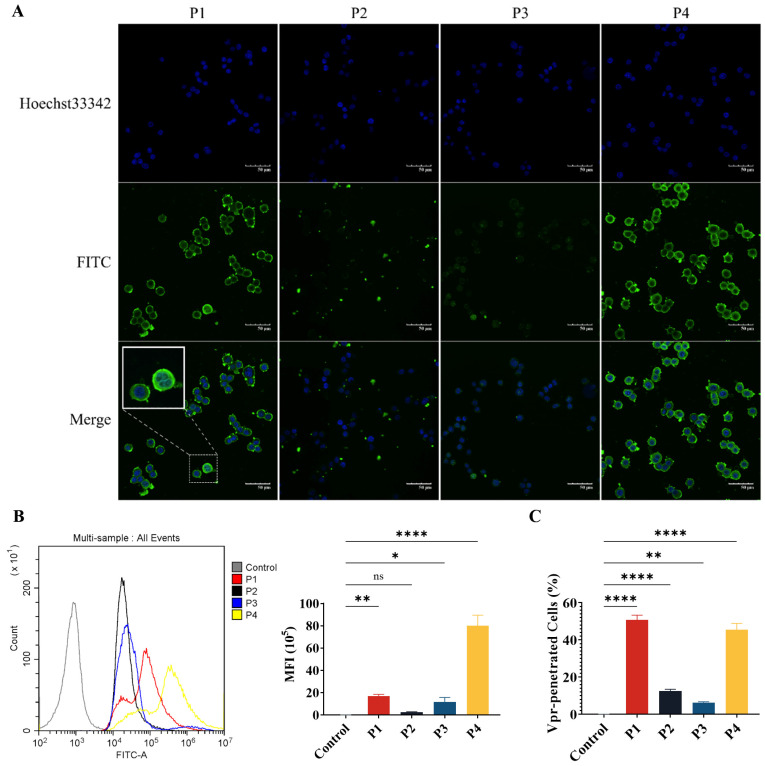
The Vpr peptides can penetrate the tumor cell membrane and enter the cells. (**A**) After being treated with 40 μM Vpr peptides (FITC labeled) for 2 h, HT-29 cells were observed using CLSM. Scale bar: 50 μm. (**B**,**C**) After being treated with 40 μM Vpr peptides (FITC labeled) for 2 h, the MFI (**B**) and Vpr-penetrated HT-29 cells (**C**) were analyzed using flow cytometry (n = 3, MFI: mean fluorescence intensity). The data are presented as means ± SD, ns: no significance, * *p* < 0.0332, ** *p* < 0.0021, **** *p* < 0.0001.

The P1, P2, and P4 Vpr peptides all contain structural domains that may be related to membrane penetration. Therefore, we have evaluated the membrane penetration ability of the Vpr peptides. FITC-labeled Vpr peptides were co-incubated with HT-29 tumor cells for 2 h, then washed and observed using a confocal laser scanning microscope (CLSM). In the P1 and P4 groups, strong FITC fluorescence signals were observed inside the cells, indicating that an abundance of P1 and P4 had penetrated the cell membrane and entered the cells, suggesting that P1 and P4 have strong membrane penetration ability. In addition to observing that P1 and P4 can enter the cells, it was also observed that P1 and P4 are highly enriched on the cell membrane (Figure 2A). Therefore, it is speculated that the Vpr peptides may insert into the tumor cell membrane and cause damage to the membrane structure, leading to the release of membrane proteins.

Next, flow cytometry was used to quantitatively analyze the mean fluorescence intensity (MFI) of FITC and the proportion of FITC^+^ Vpr-penetrated cells. The results showed that for the cells incubated with P1, the proportion of FITC^+^ cells was 97.34% and the MFI was 7.74 × 10^5^, which were significantly higher than the control group. For the P4 group, the proportion of FITC^+^ cells was 90.93% and the MFI was 5.64 × 10^6^, which were also significantly higher than the control group. Consistent with the results observed using CLSM, P1 and P4 exhibited strong membrane penetration ability. The proportion of FITC^+^ cells was only 12.57% for the P2 group and 6.317% for the P3 group, suggesting that the membrane penetration ability of P2 and P3 is much weaker than that of P1 and P4 (Figure 2B,C). These results demonstrate that P1 and P4 have membrane penetration functions and can effectively penetrate the tumor cell membrane to exert their effects inside the cells.

### 3.3. Vpr Peptides Released Tumor Antigens and Induced ICD

**Figure 3:**
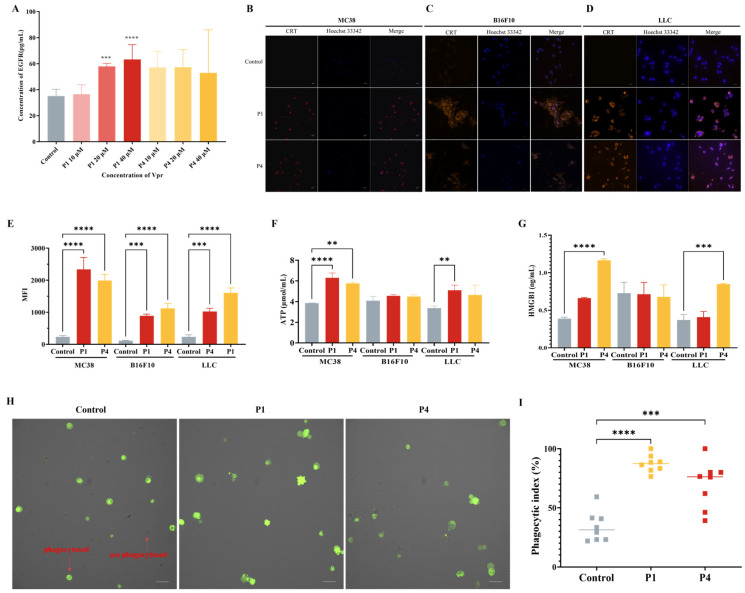
Vpr peptides released tumor antigens and induced ICD. (**A**) EGFR release was evaluated using an ELISA (n = 5). (**B**–**D**) The surface exposure of CRT on MC38 (**B**), B16F10 (**C**), and LLC (**D**) cells was evaluated using CLSM. Blue: DAPI; red: CRT. Scale bar, 50 μm. (**E**) The surface exposure of CRT was analyzed using flow cytometry (n = 3). (**F**) ATP release was evaluated using an ATP assay kit (n = 3). (**G**) HMGB1 release was evaluated using an ELISA (n = 3). (**H**,**I**) Phagocytosis test of Vpr peptides promoting macrophage Ana-1 to phagocytose LLC cells (CFDA-SE labeled), Ana-1 were observed (**H**) and counted (**I**). Green: LLC cells. Scale bar, 50 μm. The data are presented as means ± SD, ** *p* < 0.0021, *** *p* < 0.0002, **** *p* < 0.0001.

Based on the results observed using CLSM, to investigate whether Vpr peptides can induce the release of tumor antigens, we used the membrane antigen EGFR as a representative and evaluated the EGFR level in the cell culture supernatants of HT-29 tumor cells treated with Vpr peptides using an ELISA. The results showed that compared to the control group, the cell culture supernatants of the P1 and P4 treatment groups had higher levels of EGFR, indicating that P1 and P4 promoted the release of tumor cell membrane antigens (Figure 3A). To investigate whether Vpr peptides can induce ICD in tumor cells, we used three tumor cell lines with different immunogenicity, MC38, B16F10, and LLC [53], and evaluated the three hallmarks of ICD, namely, CRT, ATP, and HMGB1 after Vpr peptide treatment. The results showed that after 24 h of P1 and P4 treatment, clear CRT exposure was observed on the surface of all three tumor cell lines (Figure 3B–D). Concurrently, the CRT exposure on the tumor cell surface was also quantitatively analyzed using flow cytometry. Compared to the control group, the CRT signal on the tumor cells was significantly increased after P1 and P4 treatment (Figure 3E). The changes in the secretion level of ATP from tumor cells were evaluated using an ATP assay kit. The results showed that the ATP levels in the cell supernatants of the P1 and P4 treatment groups were significantly increased in MC38 and LLC cells, indicating that P1 and P4 can promote the secretion of ATP from tumor cells, and the effect of P1 was stronger than that of P4 (Figure 3F). The changes in the secretion of HMGB1 from tumor cells were evaluated using an ELISA. The results showed that the cell supernatants of the P1 and P4 treatment groups for MC38 and LLC cells had higher levels of HMGB1, indicating that P1 and P4 can promote the secretion of HMGB1 from MC38 and LLC tumor cells (Figure 3G). However, the ATP and HMGB1 in the culture supernatant of the B16F10 cells showed no significant changes following Vpr peptide treatment; the underlying reason for this remains to be explored.

Previous studies have shown that CRT is a major pro-phagocytic signal in various cancers [54,55,56]. Therefore, we evaluated the effect of Vpr peptide-treated LLC cells on the phagocytic activity of the macrophage cell line Ana-1 using CLSM. The results showed that after 12 h of Vpr peptide treatment of LLC cells, the proportion of macrophage Ana-1 cells phagocytosing LLC cells was significantly increased. Compared to the control group, the average phagocytic index increased from 34.0% to 87.4% in the P1 treatment group and increased to 70.0% in the P4 treatment group (Figure 3H,I). These results indicate that Vpr peptides can promote the phagocytosis of tumor cells by macrophages.

The above results indicate that the Vpr peptides P1 and P4 can induce ICD in tumor cells, suggesting their potential to release the full repertoire of tumor antigens.

### 3.4. Abscopal Anti-Tumor Effects of Vpr Peptide ISV

**Figure 4:**
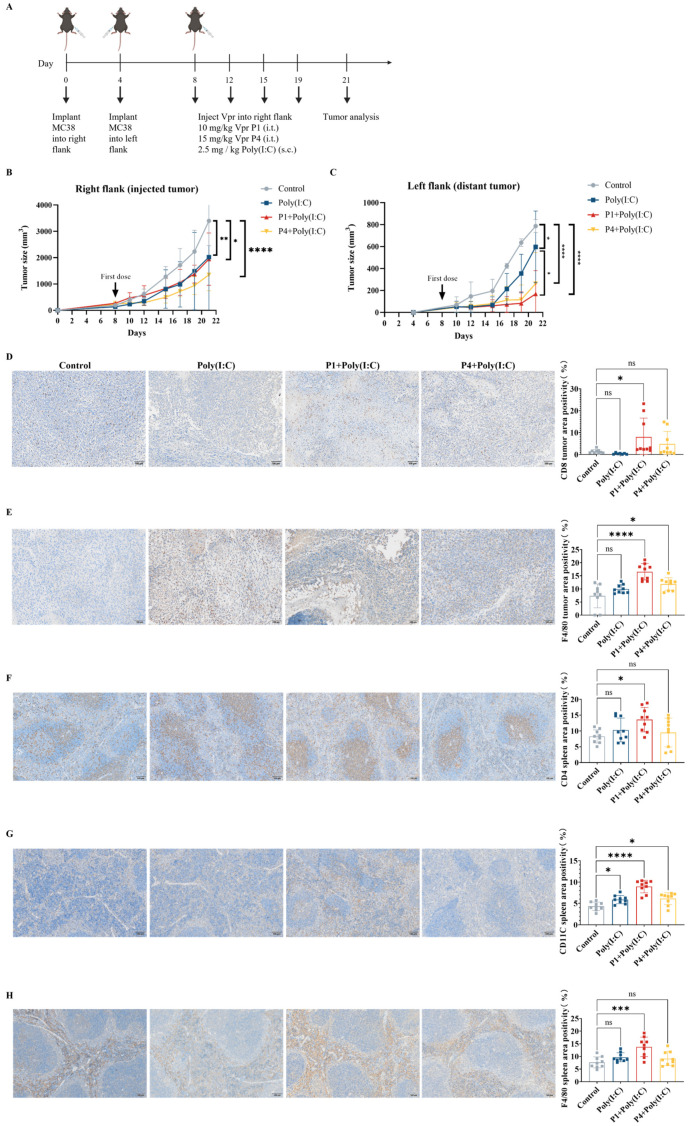
The Vpr peptide vaccine in situ inhibited MC38 tumor growth and activated anti-tumor immunity. (**A**) Treatment scheme. Mice were inoculated in the right flank with MC38 cells on day 0. After 4 days, MC38 cells were implanted into the left flank to simulate tumor metastasis. The mice were treated with intratumoral injections of 10 mg/kg P1or 15 mg/kg p4 in PBS, with a total volume of 50 μL, along with subcutaneous administration of 2.5 mg/kg of Poly(I:C) on days 8, 12, 15, and 19 (five mice per group, n = 5). Animals that died due to non-treatment-related causes prior to the study endpoints were excluded from statistical analysis. (**B**) Growth of tumors in the right flank. (**C**) Growth of tumors in the left flank. (**D**–**H**) IHC examination of CD8 (**D**), F4/80 (**E**) in right tumor tissue, and CD4 (**F**), CD11C (**G**), F4/80 (**H**) in spleen. Scale bar, 100 μm (n = 3, three scenes per sample). The data are presented as means ± SD, ns: no significance, * *p* < 0.0332, ** *p* < 0.0021, *** *p* < 0.0002, **** *p* < 0.0001.

To further validate whether the Vpr peptides P1 and P4 can be used as in situ vaccination (ISV) to stimulate systemic anti-tumor immunity, we established a mouse MC38 colorectal cancer bilateral subcutaneous tumor model. Starting on day 8 after tumor inoculation, we injected either 10 mg/kg of Vpr peptide P1 or 15 mg/kg of Vpr peptide P4 directly into the right tumor, along with subcutaneous administration of 2.5 mg/kg of Poly(I:C), twice per week (Figure 4A). Poly(I:C) was used as a TLR3 adjuvant to enhance DC maturation. P1 (MW ≈ 4.5 kDa) required 10 mg/kg and P4 (MW ≈ 6.7 kDa) required 15 mg/kg to deliver equivalent molar concentrations to ensure fair comparison of peptide efficacy. The results showed that in the MC38 colorectal cancer bilateral subcutaneous tumor model, both the P1 + Poly(I:C) in situ vaccination and the P4 + Poly(I:C) in situ vaccination exhibited good tumor growth inhibition for the right tumor (injected tumor). At 21 days after tumor inoculation, which was 13 days after the start of treatment, the right tumor growth inhibition rate was 42.82% for the P1 + Poly(I:C) in situ vaccination group and 60.32% for the P4 + Poly(I:C) in situ vaccination group (Figure 4B). To evaluate the systemic anti-tumor effects induced by in situ vaccination, in addition to administering the treatment directly into the right tumor, we also recorded the growth of the left tumor (distant tumor) to assess the abscopal effects of the vaccine. In the MC38 model, both the P1 + Poly(I:C) in situ vaccination and the P4 + Poly(I:C) in situ vaccination also exhibited significant inhibition of tumor growth for the left tumor. At 21 days after tumor inoculation, the tumor growth inhibition rate for the left tumor was 78.52% for the P1 + Poly(I:C) group and 55.54% for the P4 + Poly(I:C) group, indicating a slightly better effect for the P1 + Poly(I:C) in situ vaccination (Figure 4C). The above results demonstrate that the ISV with Vpr peptides P1 and P4 exhibited significant inhibitory effects on the MC38 tumor xenograft model. Not only were they able to suppress the growth of the tumor on the treated side, but they also elicited abscopal effects, inhibiting the growth of the contralateral tumor as well.

To evaluate the immune activation effects of the Vpr peptide, immunohistochemistry (IHC) was performed for CD8 and F4/80 on samples from the right tumors after the efficacy experiments. The results showed that the ISV with P1 + Poly(I:C) significantly increased the levels of CD8 and F4/80 in the right tumors, indicating increased infiltration of CD8^+^ T cells and macrophages. The ISV with P4 + Poly(I:C) significantly increased the F4/80 levels in the right tumors and slightly increased the CD8 levels (Figure 4D,E). To evaluate systemic immune activation, IHC was also performed for CD4, CD11c, and F4/80 on spleen samples. The results showed that the ISV with P1 + Poly(I:C) significantly increased the levels of CD4, CD11c, and F4/80 in the spleen, indicating the activation of peripheral CD4^+^ T cells, DCs, and macrophages. The ISV with P4 + Poly(I:C) significantly increased the CD11c levels in the spleen, while the CD4 and F4/80 levels did not show a significant increase (Figure 4F–H). The above results indicate that the ISV with Vpr peptide activated tumor-infiltrating T cells and macrophages, as well as peripheral T cells, DCs, and macrophages, demonstrating systemic immune activation. Specifically, the immune activation effects of P1 were more pronounced than those of P4; therefore, P1 was used for further investigation in subsequent experiments.

**Figure 5:**
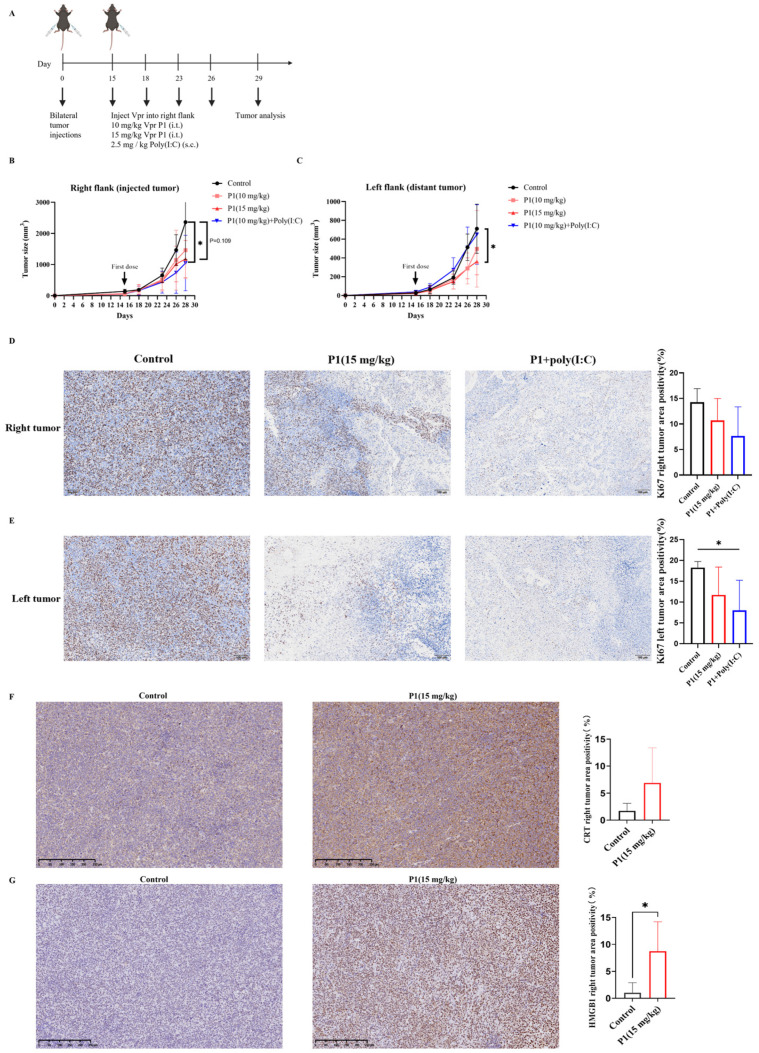
The Vpr peptide vaccine in situ inhibited LLC tumor growth. (**A**) Treatment scheme. Mice were inoculated in the right flank and left flank with LLC cells on day 0. The mice were treated with PBS, 10 mg/kg P1 or 15 mg/kg P1 (50 μL, intratumoral injection at right flank), or a combination of 10 mg/kg P1 (50 μL, intratumoral injection at right flank) and 2.5 mg/kg Poly(I:C) (subcutaneous injection) on days 15, 18, 23, and 26 (five mice per group, n = 5). Animals that died for reasons not related to administration before the endpoint were excluded from the statistics. (**B**) Growth of tumors in the right flank. (**C**) Growth of tumors in the left flank. (**D**,**E**) IHC examination of ki67 in right tumor tissue (**D**) and in left tumor tissue (**E**). Scale bar, 100 μm (n = 5). (**F**,**G**) IHC staining examination of CRT (**F**) and HMGB1 (**G**) in right tumor tissue. Scale bar, 250 μm (n = 5). (* *p* < 0.0332).

The immunogenicity of tumor cells is a key factor determining the clinical response of tumors to immunotherapy [57]. After verifying the tumor inhibition and immune activation effects of the Vpr peptide in the MC38 tumor xenograft model, to further explore whether the Vpr peptide can increase the immunogenicity of low-immunogenicity tumors and activate effective anti-tumor immune responses, we established a mouse LLC bilateral subcutaneous tumor model to evaluate the therapeutic effects of the Vpr peptide. We attempted to administer a higher dose of P1 monotherapy (15 mg/kg) and compare it with the combination of low-dose P1 (10 mg/kg) and Poly(I:C) to assess intrinsic peptide efficacy without adjuvant modulation (Figure 5A). The results showed that, compared to the control group, intratumoral injection of 15 mg/kg P1 and 10 mg/kg P1 + Poly(I:C) had similar effects, both inhibiting the growth of the right tumor (injected tumor). On day 13 after administration, the tumor inhibition rate was 50.59% for the 15 mg/kg P1 monotherapy group and 55.68% for the 10 mg/kg P1 + Poly(I:C) group (Figure 5B). As for the left tumor (distant tumor), the 15 mg/kg P1 monotherapy significantly inhibited tumor growth, with an inhibition rate of 56.75% on day 13 after administration. In contrast, the 10 mg/kg P1 + Poly(I:C) did not produce a significant tumor inhibition effect (Figure 5C). The above results indicate that in the low-immunogenicity LLC tumor xenograft model, the high-dose P1 (15 mg/kg) monotherapy and the low-dose P1 (10 mg/kg) combined with Poly(I:C) produced similar inhibitory effects on the injected tumor, while the high-dose P1 (15 mg/kg) monotherapy had better inhibitory effects on the distant tumor.

The Ki67 staining of the tumors corroborated the conclusions drawn from the tumor volume curves. Compared to the control group, both the 15 mg/kg P1 group and the 10 mg/kg P1 + Poly(I:C) group reduced the Ki67 levels in the right-side tumors, indicating suppressed proliferation of the tumor cells (Figure 5D). Furthermore, although the 10 mg/kg P1 + Poly(I:C) group did not significantly inhibit the growth of the left-side tumor, the Ki67 levels in the left-side tumor were significantly reduced, suggesting effective suppression of tumor cell proliferation (Figure 5E). The above results indicate that the high-dose P1 monotherapy and the low-dose P1 + Poly(I:C) had similar inhibitory effects on the growth of the LLC tumor xenograft model, suggesting the potential of the Vpr peptide to be used as a monotherapy to construct an anonymous antigen vaccine in situ.

It was verified that the Vpr peptide can induce ICD of tumor cells in vitro, as evidenced by the exposure of membrane-bound CRT, the release of HMGB1, and the release of ATP (Figure 3B–G). To validate whether the Vpr peptide can also induce ICD in vivo in the tumor xenograft model, we first evaluated the CRT levels in the right tumors (injected tumor) using IHC. The 15 mg/kg P1 group showed markedly increased CRT expression with membrane staining pattern in the right tumors, confirming P1’s ability to induce CRT surface exposure in vivo (Figure 5F). The IHC results for HMGB1 were consistent with those for CRT. Compared to the control group, the 15 mg/kg P1 treatment significantly increased the HMGB1 levels in the right tumors (injected tumor), with obvious diffuse positive signals in the extracellular matrix, indicating that P1 significantly increased the release of HMGB1 from tumor cells in vivo (Figure 5G). The experimental design focused on validating the in vivo ICD-inducing capability of high-dose P1 monotherapy (15 mg/kg); consequently, the IHC evaluations did not concentrate on the treatment with 10 mg/kg P1. The above results suggest that the Vpr peptide can induce ICD in the LLC tumor xenograft model, leading to the exposure of CRT on the tumor cell membranes and the release of HMGB1, thereby activating pattern recognition receptors and eliciting a potent anti-tumor immune response.

### 3.5. Systemic Immune Activation by Vpr Peptide ISV

**Figure 6:**
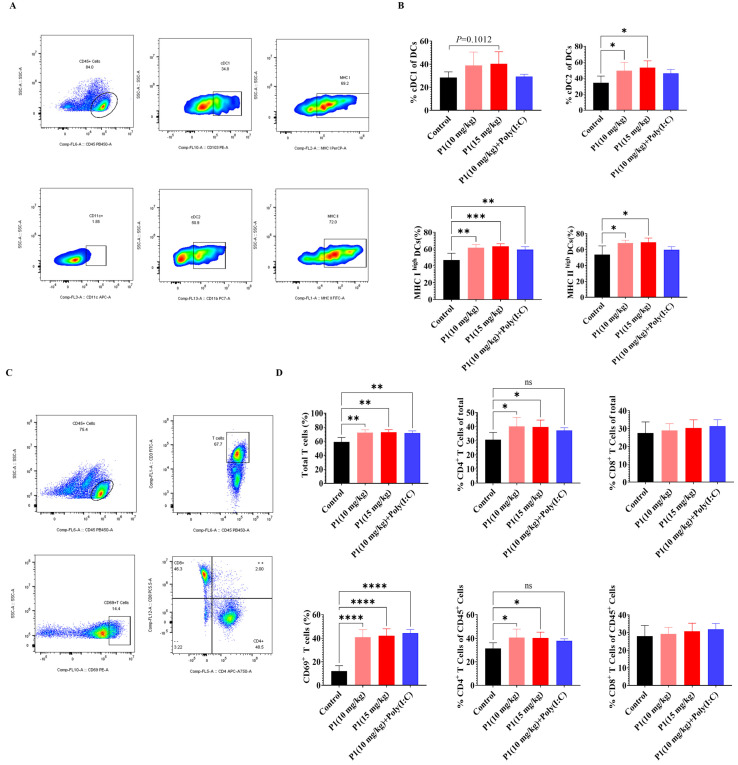
Vpr peptide-activated DCs and T cells in dLN. (**A**,**B**) The percentages of cDC1 and cDC2 in dLN were measured using flow cytometry (n = 5). (**C**,**D**) The percentages of CD4^+^ or CD8^+^T cells in dLN were measured using flow cytometry (n = 5). The data are presented as means ± SD, ns: no significance, * *p* < 0.0332, ** *p* < 0.0021, *** *p* < 0.0002, **** *p* < 0.0001.

In both the MC38 and LLC models, abscopal anti-tumor effects of the Vpr peptide were observed. To further investigate the immunological basis of this abscopal effect, we analyzed the tumor antigen presentation process using flow cytometry. The analysis of DCs in the draining lymph nodes (dLNs) of mice treated with Vpr peptide P1 showed that, compared to the control group, the proportions of cDC1 and cDC2 subsets within the DC population were increased to different extents in both the P1 monotherapy and the P1 + Poly(I:C) groups (Figure 6A,B). Additionally, the proportions of MHC I^high^ DCs and MHC II^high^ DCs were significantly elevated in the P1 (15 mg/kg) and P1 (10 mg/kg) groups, indicating that the Vpr peptide promoted the maturation of DCs in the lymph nodes and enhanced their ability to present tumor antigens (Figure 6A,B). Corresponding to the increases in cDC1 and cDC2, we also analyzed the activation status of T cells in the dLNs. The results showed that the proportions of total T cells and CD69^+^-activated T cells were significantly higher in the P1 monotherapy group and P1 + Poly(I:C) group compared to the control group, suggesting that the Vpr peptide promoted the recruitment and activation of T cells in the dLNs (Figure 6C,D). The increases in the proportions of CD4^+^ T cells were greater than those of CD8^+^ T cells, which is consistent with the more pronounced increase in cDC2 than cDC1 (Figure 6C,D). These results indicate that the ISV with Vpr peptide effectively activated DCs in the peripheral lymph nodes and enhanced their antigen presentation function, thereby activating both CD4^+^ and CD8^+^ T cells. On the other hand, macrophages also participate in the tumor antigen presentation process, so we analyzed the macrophages in the spleen and tumor. Compared to the control group, the 15 mg/kg P1 monotherapy and the P1 + Poly(I:C) groups showed increased proportions of macrophages in the spleen and both right and left tumors, with a certain degree of increase in the proportion of MHC II^+^ M1-type antigen-presenting macrophages, although the magnitude was not as significant as the increases in DCs in the dLNs (Figure S1A,B). In summary, the ISV with Vpr peptide was able to activate antigen-presenting cells, including DCs in the lymph nodes and macrophages in the tumor and periphery, by inducing tumor antigen release and ICD, thereby promoting the presentation of tumor antigens and facilitating the recruitment and activation of T cells to elicit a systemic anti-tumor immune response.

### 3.6. In Vivo Safety Evaluation of Vpr Peptide

**Figure 7:**
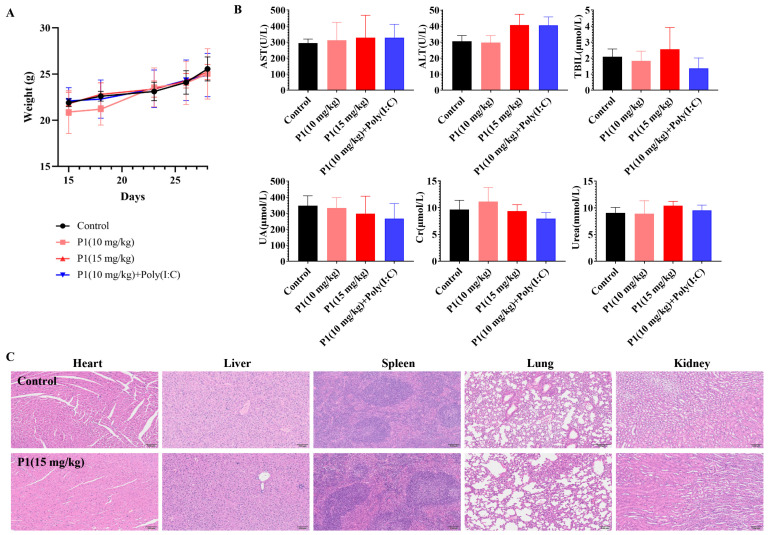
In vivo safety evaluation of Vpr peptide anonymous antigen vaccine in situ. (**A**) Mice’s weight during administration. No significant weight loss occurred (five mice per group, n = 5). (**B**) The blood biochemical indicator tests were performed for mice after administration (n = 5). There was no Vpr peptide-related toxicity observed. (**C**) H&E staining was performed on heart, liver, spleen, lung, and kidney slices after in vivo study. Samples from one mouse in each group are shown as representative. There was no drug-related damage observed in the main organs (n = 3). The data are presented as means ± SD. Scale bar: 100 μm.

After verifying the tumor inhibition and immune activation effects of the Vpr peptide, the safety of the Vpr peptide was also an important issue that needed to be addressed. We conducted a general toxicity evaluation of the Vpr peptide in situ vaccine by monitoring mouse body weight changes, histological examination of major organs, and blood biochemical indicators. The mice maintained stable weight gain throughout the dosing period, without any significant individual weight loss, preliminarily indicating that the Vpr peptide had no obvious toxicity in mice (Figure 7A). The P1 monotherapy or P1 + Poly(I:C) did not have a significant impact on the kidney function indicators (UA, CRE, URE), indicating that the Vpr peptide did not have obvious kidney toxicity (Figure 7B). While most treatment groups showed comparable AST, ALT, and BIL levels to the controls, a moderate increase in ALT was observed in the P1 15 mg/kg and P1 + adjuvant groups (Figure 7B). These values remained within the normal clinical physiological range, suggesting no significant hepatotoxicity [58]. After the dosing was completed, the major organs (heart, liver, spleen, lung, and kidney) of the mice were subjected to H&E staining to evaluate any organ damage caused by the Vpr peptide. Compared to the control group, the 15 mg/kg P1 treatment group did not show any significant tissue damage or pathological changes in the heart, liver, spleen, lung, and kidney (Figure 7C). In summary, our preliminary results provide evidence supporting the favorable safety profile of the Vpr peptide in situ vaccine. These findings suggest that the utilization of Vpr peptide for therapeutic purposes may potentially mitigate associated risks.

### 3.7. The Combination of Vpr Peptides and SIRPαFc Inhibited the Growth of the Distant Tumor

**Figure 8:**
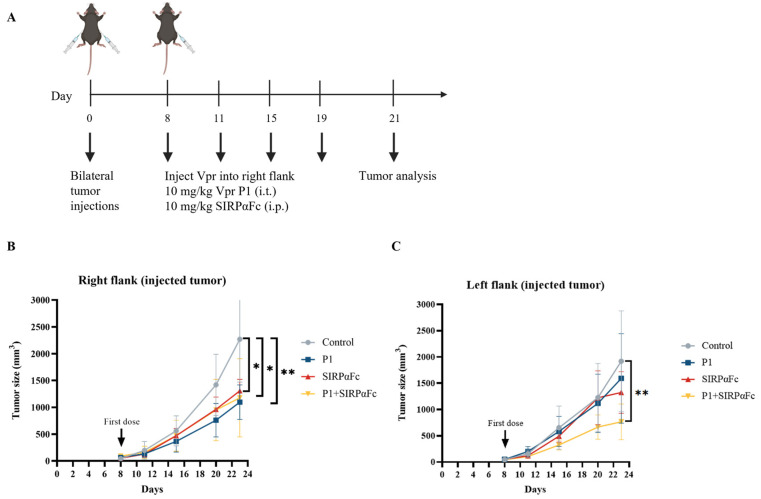
The combination of Vpr peptide and SIRPαFc inhibited the growth of the distant tumor. (**A**) Treatment scheme. Mice were inoculated in the right flank and left flank with LLC cells on day 0. The mice were treated with intratumoral injections (right flank) of 10 mg/kg P1 or/and 10 mg/kg SIRPαFc (i.p.) on days 8, 11, 15, and 19 (five mice per group, n = 5). Animals that died of causes unrelated to the administration before the experimental endpoint were excluded from the statistics. (**B**) Growth of tumors in the right flank. (**C**) Growth of tumors in the left flank. The data are presented as the mean ± SD, * *p* < 0.0332, ** *p* < 0.0021.

The immunogenicity of cancer cells is a fundamental determinant of the response to immune checkpoint inhibitors (ICIs). Tumors with very low or no immunogenicity will not respond to treatment strategies that aim to enhance the immune response. Therefore, ICIs can only be used to treat tumors with sufficient immunogenicity, and enhancing tumor immunogenicity may have the potential to convert non-responsive tumors into responsive ones [57]. Studies have shown that in various cancers, CRT is a major pro-phagocytic signal, and the increase in CRT expression on the tumor cell surface is positively correlated with the increase in CD47 expression, possibly based on a feedback regulation mechanism that balances the immune-suppressive signal mediated by CD47 and the phagocytic signal mediated by CRT [54,55,56,59]. The increase in tumor cell surface CRT is also positively correlated with the efficacy of anti-CD47 antibody-induced phagocytosis [54]. Therefore, we first examined whether the Vpr peptide affects the expression of CD47 on the tumor cell surface to explore the feasibility of combining the Vpr peptide in situ vaccine with CD47 blockade therapy. Compared to the control group, the P1 monotherapy (15 mg/kg) group showed decreased CD47 levels in the right tumor (injected tumor). We hypothesize that this may be due to the Vpr peptide disrupting the tumor cell membrane, thereby releasing CD47 from the tumor cell membrane (Figure S2A). In the left tumor (distant tumor), the P1 monotherapy (15 mg/kg) group showed a certain degree of increase in tumor CD47 levels compared to the control group (Figure S2B). Therefore, we further established the LLC bilateral subcutaneous tumor model to evaluate the anti-tumor effects of Vpr peptide combined with SIRPαFc, a fusion protein for CD47 blockade therapy [60,61,62,63,64] (Figure 8A). P1 monotherapy (10 mg/kg, i.t.), SIRPαFc monotherapy (10 mg/kg, i.p.), and their combination all significantly inhibited the growth of the right tumor (Figure 8B). For the left tumor, P1 or SIRPαFc monotherapy did not show obvious inhibitory effects on tumor growth, while the combination of P1 and SIRPαFc significantly inhibited the growth of the left tumor (Figure 8C). The above results suggest that the Vpr peptide treatment may induce the exposure of CRT and enhance the pro-phagocytic signal, leading to the upregulation of the phagocytosis-inhibitory molecule CD47 in the tumor. The combination of the Vpr peptide and the CD47 inhibitor SIRPαFc fusion protein can exert a synergistic effect, significantly inhibiting the growth of the distant tumor.

### 3.8. Proteomic Analysis to Explore the Mechanisms of Anti-Tumor Activity of Vpr Peptide

**Figure 9:**
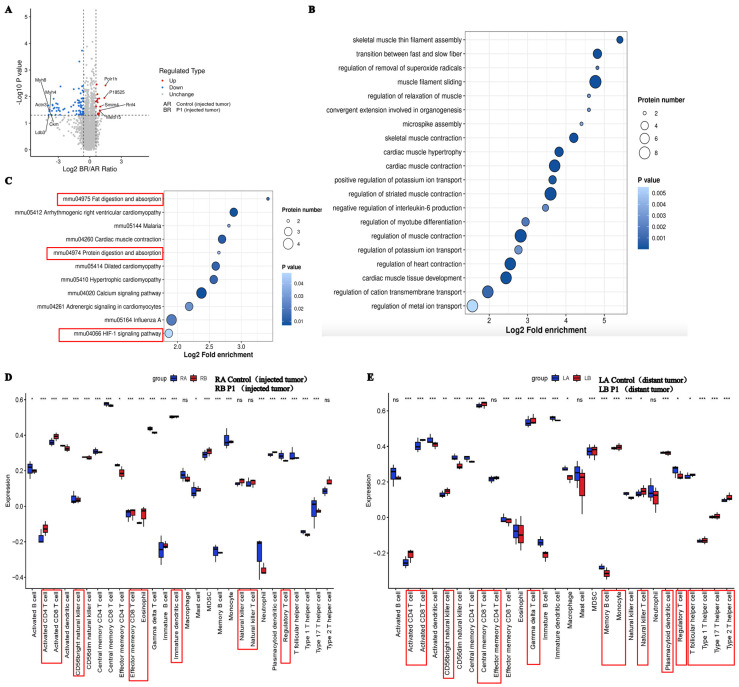
Proteomic analysis to explore the mechanism of anti-tumor activity of Vpr peptide. (**A**) Volcano plot showed the differentially expressed genes between the P1 treatment group and control group. (**B**,**C**) GO analysis (**B**) and KEGG (**C**) analysis for the genes significantly downregulated by P1 compared to the control. (**D**,**E**) The identification of immune gene sets in injected tumor (**D**) and distant tumor (**E**) using ssGSEA. * *p* < 0.05, ** *p* < 0.01, *** *p* < 0.001.

To further elucidate the underlying mechanisms of Vpr peptides exhibiting anticancer performance, we performed 4D-FastDIA proteomic analysis on the treated tumor samples. A total of 7672 proteins were identified, with 7659 being quantifiable. Differential analysis showed that, compared to the control group, P1 treatment significantly altered the expression of 89 proteins in the right-side tumor, with 17 proteins significantly upregulated and 72 proteins significantly downregulated (Figure 9A). Gene Ontology (GO) enrichment analysis of the differentially expressed proteins indicated that the downregulated proteins in the Vpr peptide treatment group were mainly cytoskeletal proteins or proteins associated with cytoskeletal functions, suggesting that the Vpr peptide may inhibit tumor cell invasion and metastasis by disrupting the maintenance of tumor cell morphology and suppressing tumor cell motility (Figure 9B). The downregulation of CD36, collagen I, and 4E-BP1 is associated with tumor suppression. Blocking CD36 can reduce regulatory T cells and increase cytotoxic T cells in the tumor, inhibiting tumor growth [65]. Collagen I knockdown can weaken cell–cell adhesion, reduce proliferation, and increase sensitivity to chemotherapeutic drugs [66]. 4E-BP1 is a predicted oncogene in many cancer cell lines and a driver of cancer cell proliferation [67]. The Vpr peptide may inhibit tumor growth through these pathways. Single-sample gene set enrichment analysis (ssGSEA) was used to estimate the relative enrichment of immune cell gene sets in the tumor samples. Compared to the control group, the Vpr peptide significantly promoted the infiltration of various immune cells, including CD4^+^ T cells, CD8^+^ T cells, NK cells, and NKT cells, in both the right tumor (injected tumor) and the left tumor (distant tumor) while reducing the infiltration of FoxP3^+^ regulatory T cells (Figure 9D,E). In summary, the Vpr peptide can exert its tumor-inhibitory effects through multiple mechanisms, including disrupting tumor cell morphology, suppressing tumor cell motility and adhesion, and promoting the infiltration of anti-tumor immune cells.

## 4. Discussion

Previous studies have shown that Vpr’s functions in inducing apoptosis, penetrating cell membranes, and nuclear localization signals depend on distinct structural domains [28,29,30,31,32], but the precise functional domains remain undefined. Our peptide designs were based on sequence combinations empirically tested in Ling Du’s prior work [51]. In this study, we designed and synthesized four Vpr peptides, including two Vpr peptides (P1 and P4) with both membrane-penetrating and apoptosis-inducing functions, one membrane-penetrating peptide (P2), and one apoptosis-inducing peptide (P3). At low concentrations, the Vpr peptides exhibited certain anti-apoptotic effects and promoted the proliferation of tumor cells, while at higher concentrations (above 1 μM), P1 and P4 caused significant tumor cell apoptosis. It was observed that during the process of Vpr peptide-induced cell apoptosis, some peptides successfully penetrated the cell membrane and entered the intracellular space, indicating that the membrane-penetrating function of Vpr was retained in the peptides. Additionally, an abundance of peptides was observed to be embedded on the cell membrane surface, and the integrity of the cell membrane was observed to be partially damaged. Therefore, we hypothesized that the Vpr peptides might be able to induce the release of tumor antigens while penetrating the cell membrane and inducing cell apoptosis. With tumor membrane protein EGFR taken as representative, the ELISA results showed that the Vpr peptides promoted the release of soluble membrane antigen proteins from tumor cells into the culture supernatant. Furthermore, we verified the functions of Vpr peptides in promoting CRT exposure, ATP, and HMGB1 release as the three hallmarks of Vpr peptide-induced tumor cell ICD. Finally, due to the correlation between CRT exposure and phagocytosis, we also semi-quantitatively verified the effect of Vpr peptides in promoting the phagocytosis of tumor cells by macrophages using CLSM. These results preliminarily confirm the feasibility of using Vpr peptide as anonymous antigen vaccine in situ.

**Figure 10:**
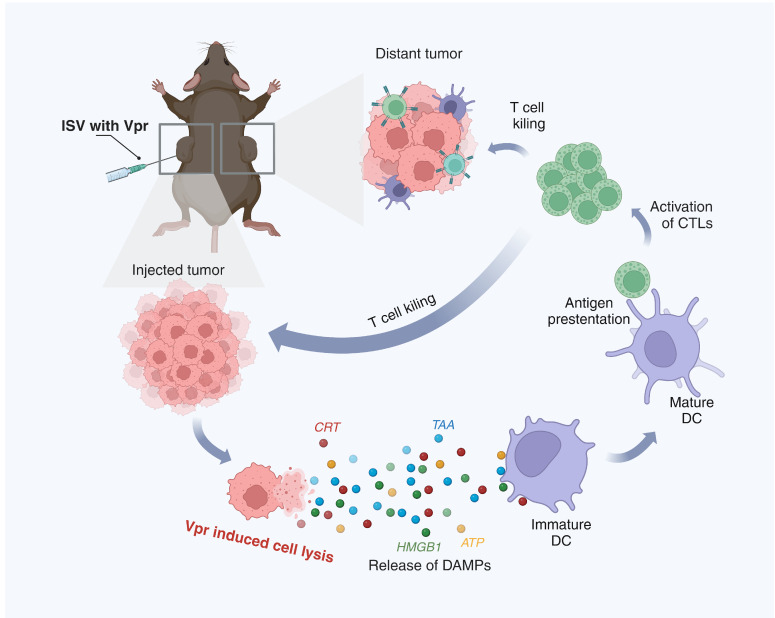
Schematic description of the mechanism of the ISV with Vpr peptides. (1) In the injected tumor, Vpr peptides induce ICD of tumor cells, characterized by surface exposure of CRT, release of HMGB1 and ATP, and liberation of TAAs. (2) The released DAMPs and TAAs promote phagocytosis of dying tumor cells by DCs and induce DC maturation. (3) Mature DCs migrate to draining lymph nodes and present tumor antigens to CD8^+^ and CD4^+^ T cells via MHC I and II molecules, respectively. (4) Activated tumor-specific T cells enter circulation and infiltrate both injected and distant tumor sites to exert anti-tumor effects. (Created in BioRender. Pan, D. (2025) https://BioRender.com/gcgn22a (accessed on 5 June 2025)).

Cancer vaccines are an emerging immunotherapy approach that utilizes tumor antigens to induce tumor-specific immune responses and exert anti-tumor functions. Anonymous antigen vaccines in situ, building upon traditional pre-defined cancer vaccines, involve vaccination at the tumor site to utilize the tumor as an antigen repertoire, inducing more specific and potent anti-tumor immune responses and avoiding the steps of identifying and obtaining tumor antigens required for pre-defined vaccines. We confirmed that the Vpr peptides P1 and P4, constructed from the membrane-penetrating and apoptosis-inducing domains of the HIV-1 viral protein R (Vpr), have the functions of releasing tumor cell antigens and inducing ICD of tumor cells. These are considered potential candidates for anonymous antigen vaccines in situ, while the specific molecular pathways through which Vpr peptides induce apoptosis remain to be fully elucidated and warrant further investigation. Subsequently, we established a bilateral MC38 tumor model to preliminarily evaluate the anti-tumor effects of Vpr peptide in situ vaccines. Regarding the dose disparity between P1 and P4 (10 mg/kg for P1 vs. 15 mg/kg for P4 in the MC38 model), this was intentionally designed to achieve equimolar concentration dosing based on molecular weight differences. Specifically, P1 (MW ≈ 4.5 kDa) required 10 mg/kg and P4 (MW ≈ 6.7 kDa) required 15 mg/kg to deliver equivalent molar concentrations. In the injected tumor, the combination of P4 + Poly(I:C) showed slightly stronger effects than P1 + Poly(I:C), which may be due to the additional apoptosis-inducing domain in P4 leading to stronger direct induction of tumor cell apoptosis. In the distant tumor, the P1 vaccine showed slightly stronger effects than P4. The inhibition of the distant tumor indicates that the ISV with Vpr peptide successfully induced a systemic anti-tumor immune response, so we further examined the activation of intratumoral and peripheral immune cells. The results showed that the immune activation effects induced by the P1 vaccine were stronger than P4, whether in terms of intratumoral CD8^+^ T cells and macrophages or splenic CD4^+^ T cells, DCs, and macrophages. This may be because the moderate apoptosis-inducing function of P1 is more suitable for activating anti-tumor immunity. Therefore, in subsequent studies, we selected P1 as the candidate for the anonymous antigen vaccine in situ. We acknowledge the theoretical possibility that Vpr peptides might induce distant effects through systemic migration; however, definitive confirmation would require further trafficking studies with labeled peptides. To investigate the immunomodulatory effect of P1 monotherapy, a P1 monotherapy vaccine group was included in the efficacy study using the low-immunogenic LLC transplanted tumor model and compared with the P1 + Poly(I:C) vaccine. For the injected tumor, the high-dose P1 monotherapy vaccine and the low-dose P1 + Poly(I:C) vaccine showed similar tumor inhibition effects. For the distant tumor, the low-dose P1 + Poly(I:C) vaccine failed to significantly inhibit the growth of the distant low-immunogenic LLC tumor, while the high-dose P1 monotherapy vaccine significantly inhibited tumor growth. IHC and flow cytometry results also showed that the high-dose P1 monotherapy vaccine induced significant ICD of tumor cells and enhanced the antigen presentation function of lymph node DCs, thereby recruiting and activating T cells. The P1 monotherapy vaccine increased the proportion of cDC1 and cDC2 subsets in the lymph nodes, which presented antigens to CD8^+^ T cells and CD4^+^ T cells, respectively, promoting the activation and differentiation of T cells in the dLN and generating a potent anti-tumor immune response [68,69,70,71,72]. The activated T cells then circulated to various tumor lesions to exert systemic anti-tumor effects [73]. The mechanism described above for the in situ vaccine effect of Vpr peptides is shown in detail in Figure 10. The anti-tumor mechanisms of the Vpr peptide were further explored through proteomic analysis, summarizing its anti-tumor effects in terms of disrupting tumor cell morphology, inhibiting tumor cell motility, inhibiting cell adhesion, and promoting immune cell infiltration. Furthermore, mouse body weight, blood biochemical indicators, and H&E staining of major organs also indicated good safety of the P1 monotherapy vaccine. These results suggest that the Vpr peptide P1 exhibits potential for development as a novel anonymous antigen vaccine in situ with a favorable safety profile. We acknowledge that the experimental group design in this animal study could be optimized. For instance, the MC38 model lacked monotherapy groups for P1 and P4, which may compromise the evaluation of their individual therapeutic efficacy. Similarly, the LLC model did not include a Poly(I:C) monotherapy group, potentially affecting the attribution analysis of the abscopal effect. These design choices were made with animal welfare considerations (to reduce redundant groups) but might compromise the clarity of the conclusions. Future studies will employ a factorial design to systematically analyze the independent contributions of peptides, adjuvants, and combination therapies. While our data demonstrate potent T cell-mediated antitumor immunity, the potential contribution of humoral responses warrants consideration. P4 peptide may lead to antibody induction, and such antibodies could theoretically engage FcγR-expressing effector cells (e.g., NK cells, macrophages) to eliminate tumor cells via ADCC/ADCP [74]. Future studies should directly quantify anti-peptide antibodies and test their cytotoxicity against tumors cells in complement deposition assays.

Studies have shown that the increased phagocytic signal caused by CRT exposure may upregulate the expression of CD47, the immune checkpoint molecule that is the main inhibitory signal for regulating phagocytosis [54]. And, we found that the ISV with Vpr peptide can significantly induce exposure of calreticulin (CRT) on the tumor cell membrane, promoting the phagocytosis of tumor cells by macrophages. Additionally, the IHC results revealed that the Vpr peptide vaccine led to upregulation of CD47 expression in the distant tumor. Therefore, we attempted to combine the Vpr peptide vaccine with the CD47 immune checkpoint inhibitor SIRPαFc. In our previous study, we constructed a novel CD47-targeting fusion protein SIRPαFc, which was found to increase the phagocytic and cytotoxic activities of macrophages against NSCLC cells [60,61,62,63,64]. We found that the combination therapy of the Vpr peptide vaccine and SIRαFc showed significantly better inhibition of the distant tumor compared to low-dose P1 monotherapy vaccine or SIRPαFc monotherapy.

In summary, we constructed a novel Vpr peptide drug, P1, using the Vpr 60–92 sequence, which has not been previously reported as a candidate cancer vaccine. Our investigations suggest that Vpr peptide P1 may induce tumor antigen release and trigger immunogenic cell death (ICD) in tumors. Preliminary results from transplanted tumor models indicate potential tumor-suppressive effects and immune-activating functions. The Vpr peptide vaccine exhibits the characteristic of inducing a systemic immune response to attack distant tumor lesions with injection at a single tumor site. By releasing tumor antigens to activate an anti-tumor immune response, it is expected to have patient-specific efficacy. This approach also minimizes the technical resources required for ex vivo vaccine development, facilitating rapid vaccine development. Furthermore, the ISV with Vpr peptide promotes the release of the whole tumor cell (WTC) antigen and induces tumor immunogenic cell death (ICD). Consequently, despite the impact of intratumoral heterogeneity (ITH), it effectively enhances the intratumoral infiltration of immune cells, induces a robust anti-tumor immune response, and minimizes the risk of tumor immune escape. Therefore, it represents a promising candidate for anonymous antigen vaccine. The ISV of Vpr peptide offers access to all cellular antigens at levels that accurately reflect their presence within the cell by changing tumor cells to stressed and dying cells that initiate ‘natural immunity’. This approach avoids any observer selection or bias, potentially offering a more precise representation of an individual’s tumor than current technologies can achieve. Overall, the Vpr peptide ISV offers several distinct advantages, including: (1) the ability to utilize the complete repertoire of tumor antigens rather than being limited to predefined epitopes; (2) greater patient specificity through recognition of unique neoantigens; and (3) dramatically reduced production time since it does not require extensive antigen screening. However, we also acknowledge the limitations of in situ administration, particularly regarding accessibility of deep-seated tumors and potential heterogeneity in drug distribution. To address these challenges, we are currently exploring combination strategies with penetration enhancers and nanodelivery techniques.

## 5. Conclusions

In conclusion, we designed and synthesized Vpr peptides that retain the key functions of the full-length Vpr protein, including membrane penetration and apoptosis induction. These peptides, particularly P1, demonstrated potential as anonymous antigen vaccines in situ for cancer immunotherapy. P1 and P4 effectively induced tumor cell apoptosis, antigen release, and immunogenic cell death (ICD). In animal models, P1 showed promising anti-tumor effects both as a monotherapy and in combination with immune adjuvants, enhancing antigen presentation by DCs and activating T cell responses, leading to systemic anti-tumor immunity. Notably, combining the Vpr peptide vaccine with a CD47 immune checkpoint inhibitor (SIRPαFc) further enhanced anti-tumor effects, particularly against distant tumors. Proteomic analysis revealed multiple anti-tumor mechanisms of the Vpr peptide, including disruption of tumor cell morphology and promotion of immune cell infiltration. Our study provides a novel approach for cancer immunotherapy using Vpr peptide-based vaccines, which holds promise for becoming a broad-spectrum anti-tumor strategy. Further research is warranted to optimize the therapeutic effects and explore potential combinations with other immunotherapies.

## Figures and Tables

**Table 1:** The polypeptides and sequences used in the experiment.

Peptide Designation	Amino Acid No. of Vpr (Accession Number: P05928)	Amino Acid Sequence
P1	60–92	IIRILQQLLFIHFRIGCRHSRIGVTRQRRARNG
P2	60–75	IIRILQQLLFIHFRIG
P3	71–92	HFRIGCRHSRIGVTRQRRARNG
P4	23–37 + 55–91	LEELKNEAVRHFPRIAGVEAIIRILQQLLFIHFRIGCRHSRIGVTRQRRARN

## Data Availability

All data relevant to the study are included in the article. Additional data are available upon reasonable request made to the corresponding author, L.Y.

## Supplementary Materials

The following supporting information can be downloaded at: https://www.mdpi.com/article/10.3390/vaccines13070710/s1, Figure S1: Vpr peptide activated macrophages in the tumor and periphery; Figure S2: (A,B) IHC examination of CD47 in right tumor tissue (A) and in left tumor tissue (B).

## Abbreviations

Vpr: viral protein R; CFDA SE: Carboxyfluorescein diacetate succinimidyl ester; ICD: immunogenic cell death; CRT: calreticulin; HMGB1: high mobility group protein B1; ISV: in situ vaccination; MHC: major histocompatibility complex; EGFR: epidermal growth factor receptor; ELISA: enzyme-linked immunosorbent assay; DCs: dendritic cells; H&E: hematoxylin and eosin; IHC: immunohistochemistry; SIRPα: signal regulatory protein alpha; CD47: cluster of differentiation 47; LLC: Lewis lung carcinoma; ICI: immune checkpoint inhibitor.

## References

[1] Sung H., Ferlay J., Siegel R.L., Laversanne M., Soerjomataram I., Jemal A., Bray F. (2021). Global Cancer Statistics 2020: GLOBOCAN Estimates of Incidence and Mortality Worldwide for 36 Cancers in 185 Countries. CA Cancer J. Clin..

[2] Matsiko A. (2018). Cancer immunotherapy making headway. Nat. Mater..

[3] Riley R.S., June C.H., Langer R., Mitchell M.J. (2019). Delivery technologies for cancer immunotherapy. Nat. Rev. Drug Discov..

[4] Elsheikh R., Makram A.M., Huy N.T. (2023). Therapeutic Cancer Vaccines and Their Future Implications. Vaccines.

[5] Liu J., Fu M., Wang M., Wan D., Wei Y., Wei X. (2022). Cancer vaccines as promising immuno-therapeutics: Platforms and current progress. J. Hematol. Oncol..

[6] Rojas L.A., Sethna Z., Soares K.C., Olcese C., Pang N., Patterson E., Lihm J., Ceglia N., Guasp P., Chu A. (2023). Personalized RNA neoantigen vaccines stimulate T cells in pancreatic cancer. Nature.

[7] Lin M.J., Svensson-Arvelund J., Lubitz G.S., Marabelle A., Melero I., Brown B.D., Brody J.D. (2022). Cancer vaccines: The next immunotherapy frontier. Nat. Cancer.

[8] Ren D., Xiong S., Ren Y., Yang X., Zhao X., Jin J., Xu M., Liang T., Guo L., Weng L. (2024). Advances in therapeutic cancer vaccines: Harnessing immune adjuvants for enhanced efficacy and future perspectives. Comput. Struct. Biotechnol. J..

[9] Pan D., Liu J., Huang X., Wang S., Kuerban K., Yan Y., Zhu Y.Z., Ye L. (2024). Challenges and New Directions in Therapeutic Cancer Vaccine Development. Vaccines.

[10] Saxena M., van der Burg S.H., Melief C.J.M., Bhardwaj N. (2021). Therapeutic cancer vaccines. Nat. Rev. Cancer.

[11] Saleh R., Elkord E. (2020). Acquired resistance to cancer immunotherapy: Role of tumor-mediated immunosuppression. Semin. Cancer Biol..

[12] Jhunjhunwala S., Hammer C., Delamarre L. (2021). Antigen presentation in cancer: Insights into tumour immunogenicity and immune evasion. Nat. Rev. Cancer.

[13] Mariathasan S., Turley S.J., Nickles D., Castiglioni A., Yuen K., Wang Y., Kadel E.E., Koeppen H., Astarita J.L., Cubas R. (2018). TGFβ attenuates tumour response to PD-L1 blockade by contributing to exclusion of T cells. Nature.

[14] Wu H., Fu X., Zhai Y., Gao S., Yang X., Zhai G. (2021). Development of Effective Tumor Vaccine Strategies Based on Immune Response Cascade Reactions. Adv. Healthc. Mater..

[15] Wu Y.M., Cieślik M., Lonigro R.J., Vats P., Reimers M.A., Cao X., Ning Y., Wang L., Kunju L.P., de Sarkar N. (2018). Inactivation of CDK12 Delineates a Distinct Immunogenic Class of Advanced Prostate Cancer. Cell.

[16] Strønen E., Toebes M., Kelderman S., van Buuren M.M., Yang W., van Rooij N., Donia M., Böschen M.L., Lund-Johansen F., Olweus J. (2016). Targeting of cancer neoantigens with donor-derived T cell receptor repertoires. Science.

[17] Hammerich L., Bhardwaj N., Kohrt H.E., Brody J.D. (2016). In situ vaccination for the treatment of cancer. Immunotherapy.

[18] Sellars M.C., Wu C.J., Fritsch E.F. (2022). Cancer vaccines: Building a bridge over troubled waters. Cell.

[19] Kroemer G., Galluzzi L., Kepp O., Zitvogel L. (2013). Immunogenic cell death in cancer therapy. Annu. Rev. Immunol..

[20] Krysko D.V., Garg A.D., Kaczmarek A., Krysko O., Agostinis P., Vandenabeele P. (2012). Immunogenic cell death and DAMPs in cancer therapy. Nat. Rev. Cancer.

[21] Tang R., Xu J., Zhang B., Liu J., Liang C., Hua J., Meng Q., Yu X., Shi S. (2020). Ferroptosis, necroptosis, and pyroptosis in anticancer immunity. J. Hematol. Oncol..

[22] Chiang C.L., Coukos G., Kandalaft L.E. (2015). Whole Tumor Antigen Vaccines: Where Are We?. Vaccines.

[23] Yang A., Bai Y., Dong X., Ma T., Zhu D., Mei L., Lv F. (2021). Hydrogel/nanoadjuvant-mediated combined cell vaccines for cancer immunotherapy. Acta Biomater..

[24] Kepp O., Senovilla L., Vitale I., Vacchelli E., Adjemian S., Agostinis P., Apetoh L., Aranda F., Barnaba V., Bloy N. (2014). Consensus guidelines for the detection of immunogenic cell death. Oncoimmunology.

[25] Kroemer G., Galassi C., Zitvogel L., Galluzzi L. (2022). Immunogenic cell stress and death. Nat. Immunol..

[26] Márquez-Rodas I., Longo F., Rodriguez-Ruiz M.E., Calles A., Ponce S., Jove M., Rubio-Viqueira B., Perez-Gracia J.L., Gómez-Rueda A., López-Tarruella S. (2020). Intratumoral nanoplexed poly I:C BO-112 in combination with systemic anti-PD-1 for patients with anti-PD-1-refractory tumors. Sci. Transl. Med..

[27] Vitale I., Yamazaki T., Wennerberg E., Sveinbjørnsson B., Rekdal Ø., Demaria S., Galluzzi L. (2021). Targeting Cancer Heterogeneity with Immune Responses Driven by Oncolytic Peptides. Trends Cancer.

[28] Andersen J.L., Le Rouzic E., Planelles V. (2008). HIV-1 Vpr: Mechanisms of G2 arrest and apoptosis. Exp. Mol. Pathol..

[29] Fukumori T., Akari H., Yoshida A., Fujita M., Koyama A.H., Kagawa S., Adachi A. (2000). Regulation of cell cycle and apoptosis by human immunodeficiency virus type 1 Vpr. Microbes Infect..

[30] Sherman M.P., Schubert U., Williams S.A., de Noronha C.M., Kreisberg J.F., Henklein P., Greene W.C. (2002). HIV-1 Vpr displays natural protein-transducing properties: Implications for viral pathogenesis. Virology.

[31] Kübler J., Kirschner S., Hartmann L., Welzel G., Engelhardt M., Herskind C., Veldwijk M.R., Schultz C., Felix M., Glatting G. (2016). The HIV-derived protein Vpr52-96 has anti-glioma activity in vitro and in vivo. Oncotarget.

[32] Mahalingam S., MacDonald B., Ugen K.E., Ayyavoo V., Agadjanyan M.G., Williams W.V., Weiner D.B. (1997). In vitro and in vivo tumor growth suppression by HIV-1 Vpr. DNA Cell Biol..

[33] Wallet C., Rohr O., Schwartz C. (2020). Evolution of a concept: From accessory protein to key virulence factor, the case of HIV-1 Vpr. Biochem. Pharmacol..

[34] Mbita Z., Hull R., Dlamini Z. (2014). Human immunodeficiency virus-1 (HIV-1)-mediated apoptosis: New therapeutic targets. Viruses.

[35] Brenner C., Subramaniam K., Pertuiset C., Pervaiz S. (2011). Adenine nucleotide translocase family: Four isoforms for apoptosis modulation in cancer. Oncogene.

[36] Li L., Wang C., Wen Y., Hu Y., Xie Y., Xu M., Liang M., Liu W., Liu L., Wu Y. (2018). ERK1/2 and the Bcl-2 Family Proteins Mcl-1, tBid, and Bim Are Involved in Inhibition of Apoptosis During Persistent Chlamydia psittaci Infection. Inflammation.

[37] Morellet N., Roques B.P., Bouaziz S. (2009). Structure-function relationship of Vpr: Biological implications. Curr. HIV Res..

[38] Fabryova H., Strebel K. (2019). Vpr and Its Cellular Interaction Partners: R We There Yet?. Cells.

[39] Muthumani K., Choo A.Y., Hwang D.S., Ugen K.E., Weiner D.B. (2004). HIV-1 Vpr: Enhancing sensitivity of tumors to apoptosis. Curr. Drug Deliv..

[40] Muthumani K., Lambert V.M., Sardesai N.Y., Kim J.J., Heller R., Weiner D.B., Ugen K.E. (2009). Analysis of the potential for HIV-1 Vpr as an anti-cancer agent. Curr. HIV Res..

[41] Stewart S.A., Poon B., Jowett J.B., Xie Y., Chen I.S. (1999). Lentiviral delivery of HIV-1 Vpr protein induces apoptosis in transformed cells. Proc. Natl. Acad. Sci. USA.

[42] Ma B., Zhang H., Wang J., Zhang B., Xu X., Cheng B. (2012). HIV-1 viral protein R (Vpr) induction of apoptosis and cell cycle arrest in multidrug-resistant colorectal cancer cells. Oncol. Rep..

[43] Muthumani K., Lambert V.M., Shanmugam M., Thieu K.P., Choo A.Y., Chung J.C.W., Satishchandran A., Kim J.J., Weiner D.B., Ugen K.E. (2009). Anti-tumor activity mediated by protein and peptide transduction of HIV viral protein R (Vpr). Cancer Biol. Ther..

[44] Muthumani K., Zhang D., Hwang D.S., Kudchodkar S., Dayes N.S., Desai B.M., Malik A.S., Yang J.S., Chattergoon M.A., Maguire H.C. (2002). Adenovirus encoding HIV-1 Vpr activates caspase 9 and induces apoptotic cell death in both p53 positive and negative human tumor cell lines. Oncogene.

[45] Zhang Y., Yuan J., Zhang H.Y., Simayi D., Li P.D., Wang Y.H., Li F., Zhang W.J. (2012). Natural resistance to apoptosis correlates with resistance to chemotherapy in colorectal cancer cells. Clin. Exp. Med..

[46] Berglez J.M., Castelli L.A., Sankovich S.A., Smith S.C., Curtain C.C., Macreadie I.G. (1999). Residues within the HFRIGC sequence of HIV-1 vpr involved in growth arrest activities. Biochem. Biophys. Res. Commun..

[47] Yao S., Torres A.M., Azad A.A., Macreadie I.G., Norton R.S. (1998). Solution structure of peptides from HIV-1 Vpr protein that cause membrane permeabilization and growth arrest. J. Pept. Sci..

[48] Roumier T., Vieira H.L., Castedo M., Ferri K.F., Boya P., Andreau K., Druillennec S., Joza N., Penninger J.M., Roques B. (2002). The C-terminal moiety of HIV-1 Vpr induces cell death via a caspase-independent mitochondrial pathway. Cell Death Differ..

[49] Moon H.S., Yang J.S. (2006). Role of HIV Vpr as a regulator of apoptosis and an effector on bystander cells. Mol. Cells.

[50] Godet A.N., Guergnon J., Croset A., Cayla X., Falanga P.B., Colle J.H., Garcia A. (2010). PP2A1 binding, cell transducing and apoptotic properties of Vpr(77-92): A new functional domain of HIV-1 Vpr proteins. PLoS ONE.

[51] Du L., Wu C.-S., Sun J., Yu T., Lyu P.-P., Han S.-F., Qiu C., Meng Z.-F. (2021). Multifactorial role of HIV-Vpr in cell apoptosis revealed by a naturally truncated 54aa variant. Chin. Med. J..

[52] Ayyavoo V., Mahboubi A., Mahalingam S., Ramalingam R., Kudchodkar S., Williams W.V., Green D.R., Weiner D.B. (1997). HIV-1 Vpr suppresses immune activation and apoptosis through regulation of nuclear factor kappa B. Nat. Med..

[53] Ringel A.E., Drijvers J.M., Baker G.J., Catozzi A., García-Cañaveras J.C., Gassaway B.M., Miller B.C., Juneja V.R., Nguyen T.H., Joshi S. (2020). Obesity Shapes Metabolism in the Tumor Microenvironment to Suppress Anti-Tumor Immunity. Cell.

[54] Chao M.P., Jaiswal S., Weissman-Tsukamoto R., Alizadeh A.A., Gentles A.J., Volkmer J., Weiskopf K., Willingham S.B., Raveh T., Park C.Y. (2010). Calreticulin is the dominant pro-phagocytic signal on multiple human cancers and is counterbalanced by CD47. Sci. Transl. Med..

[55] Liu C.C., Leclair P., Pedari F., Vieira H., Monajemi M., Sly L.M., Reid G.S., Lim C.J. (2019). Integrins and ERp57 Coordinate to Regulate Cell Surface Calreticulin in Immunogenic Cell Death. Front. Oncol..

[56] Guilbaud E., Kroemer G., Galluzzi L. (2023). Calreticulin exposure orchestrates innate immunosurveillance. Cancer Cell.

[57] Wang S., He Z., Wang X., Li H., Liu X.S. (2019). Antigen presentation and tumor immunogenicity in cancer immunotherapy response prediction. eLife.

[58] Mazzaccara C., Labruna G., Cito G., Scarfò M., De Felice M., Pastore L., Sacchetti L. (2008). Age-Related Reference Intervals of the Main Biochemical and Hematological Parameters in C57BL/6J, 129SV/EV and C3H/HeJ Mouse Strains. PLoS ONE.

[59] Feng M., Jiang W., Kim B.Y.S., Zhang C.C., Fu Y.X., Weissman I.L. (2019). Phagocytosis checkpoints as new targets for cancer immunotherapy. Nat. Rev. Cancer.

[60] Zhang X., Fan J., Wang S., Li Y., Wang Y., Li S., Luan J., Wang Z., Song P., Chen Q. (2017). Targeting CD47 and Autophagy Elicited Enhanced Antitumor Effects in Non-Small Cell Lung Cancer. Cancer Immunol. Res..

[61] Zhang X., Chen W., Fan J., Wang S., Xian Z., Luan J., Li Y., Wang Y., Nan Y., Luo M. (2018). Disrupting CD47-SIRPα axis alone or combined with autophagy depletion for the therapy of glioblastoma. Carcinogenesis.

[62] Zhang X., Wang Y., Fan J., Chen W., Luan J., Mei X., Wang S., Li Y., Ye L., Li S. (2019). Blocking CD47 efficiently potentiated therapeutic effects of anti-angiogenic therapy in non-small cell lung cancer. J. Immunother. Cancer.

[63] Liu J., Meng Z., Xu T., Kuerban K., Wang S., Zhang X., Fan J., Ju D., Tian W., Huang X. (2022). A SIRPαFc Fusion Protein Conjugated With the Collagen-Binding Domain for Targeted Immunotherapy of Non-Small Cell Lung Cancer. Front. Immunol..

[64] Liu J., Xu T., Pan D., Fan J., Fu Y., Huang X., Zhao W., Dong X., Zhang S., Kuerban K. (2023). A collagen-binding SIRPαFc fusion protein for targeted cancer immunotherapy. Int. Immunopharmacol..

[65] Ma X., Xiao L., Liu L., Ye L., Su P., Bi E., Wang Q., Yang M., Qian J., Yi Q. (2021). CD36-mediated ferroptosis dampens intratumoral CD8(+) T cell effector function and impairs their antitumor ability. Cell Metab..

[66] Chen Y., Yang S., Tavormina J., Tampe D., Zeisberg M., Wang H., Mahadevan K.K., Wu C.J., Sugimoto H., Chang C.C. (2022). Oncogenic collagen I homotrimers from cancer cells bind to α3β1 integrin and impact tumor microbiome and immunity to promote pancreatic cancer. Cancer Cell.

[67] Rutkovsky A.C., Yeh E.S., Guest S.T., Findlay V.J., Muise-Helmericks R.C., Armeson K., Ethier S.P. (2019). Eukaryotic initiation factor 4E-binding protein as an oncogene in breast cancer. BMC Cancer.

[68] Gross D.A., Leborgne C., Chappert P., Masurier C., Leboeuf M., Monteilhet V., Boutin S., Lemonnier F.A., Davoust J., Kichler A. (2019). Induction of tumor-specific CTL responses using the C-terminal fragment of Viral protein R as cell penetrating peptide. Sci. Rep..

[69] Collin M., Bigley V. (2018). Human dendritic cell subsets: An update. Immunology.

[70] Noubade R., Majri-Morrison S., Tarbell K.V. (2019). Beyond cDC1: Emerging Roles of DC Crosstalk in Cancer Immunity. Front. Immunol..

[71] Martín P., Ruiz S.R., del Hoyo G.M., Anjuère F., Vargas H.H., López-Bravo M., Ardavín C. (2002). Dramatic increase in lymph node dendritic cell number during infection by the mouse mammary tumor virus occurs by a CD62L-dependent blood-borne DC recruitment. Blood.

[72] Schenkel J.M., Herbst R.H., Canner D., Li A., Hillman M., Shanahan S.L., Gibbons G., Smith O.C., Kim J.Y., Westcott P. (2021). Conventional type I dendritic cells maintain a reservoir of proliferative tumor-antigen specific TCF-1(+) CD8(+) T cells in tumor-draining lymph nodes. Immunity.

[73] Miller H.L., Andhey P.S., Swiecki M.K., Rosa B.A., Zaitsev K., Villani A.C., Mitreva M., Artyomov M.N., Gilfillan S., Cella M. (2021). Altered ratio of dendritic cell subsets in skin-draining lymph nodes promotes Th2-driven contact hypersensitivity. Proc. Natl. Acad. Sci. USA.

[74] Feola S., Hamdan F., Russo S., Chiaro J., Fusciello M., Feodoroff M., Antignani G., D’Alessio F., Mölsä R., Stigzelius V. (2024). Novel peptide-based oncolytic vaccine for enhancement of adaptive antitumor immune response via co-engagement of innate Fcγ and Fcα receptors. J. Immunother. Cancer.

