# Supplementary material for: In Situ Vaccination with a Vpr-Derived Peptide Elicits Systemic Antitumor Immunity by Improving Tumor Immunogenicity
Source: Vaccines (Basel). 2025 Jun 30;13(7):710. doi: 10.3390/vaccines13070710 (PMC12298452; doi:10.3390/vaccines13070710)
Supplement: Supplementary file 1 [file vaccines-13-00710-s001.zip › vaccines-3640599-supplementary.pdf]

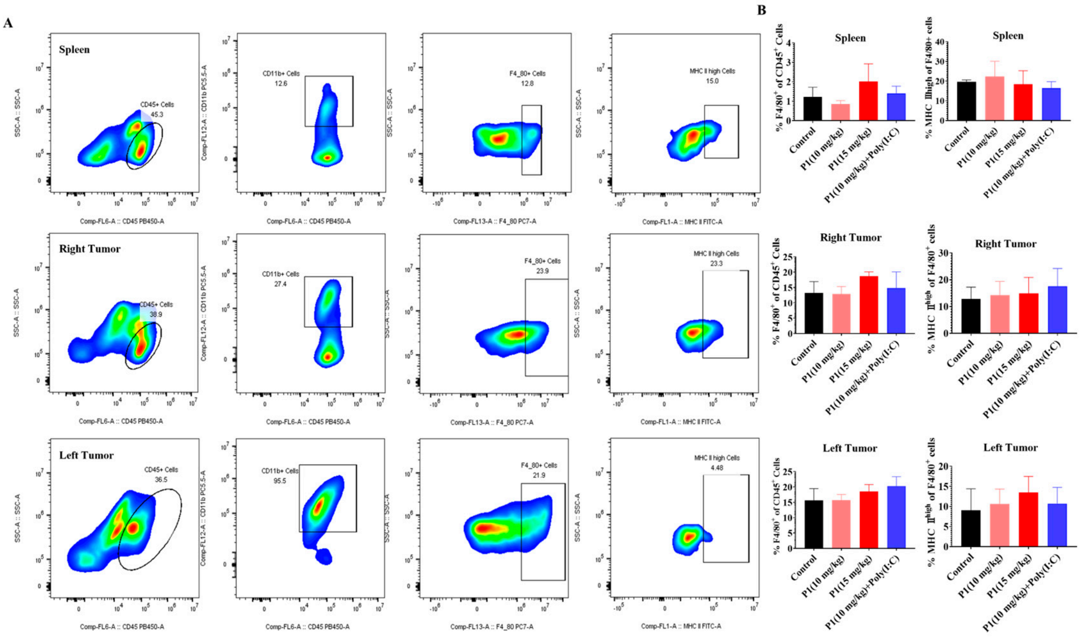

Figure S1. Vpr peptide activated macrophages in the tumor and periphery. (**A**,**B**) The percentages of macrophages in the spleen, right tumor and left tumor were measured using flow cytometry (n = 5). (The data are presented as the mean ± SD).

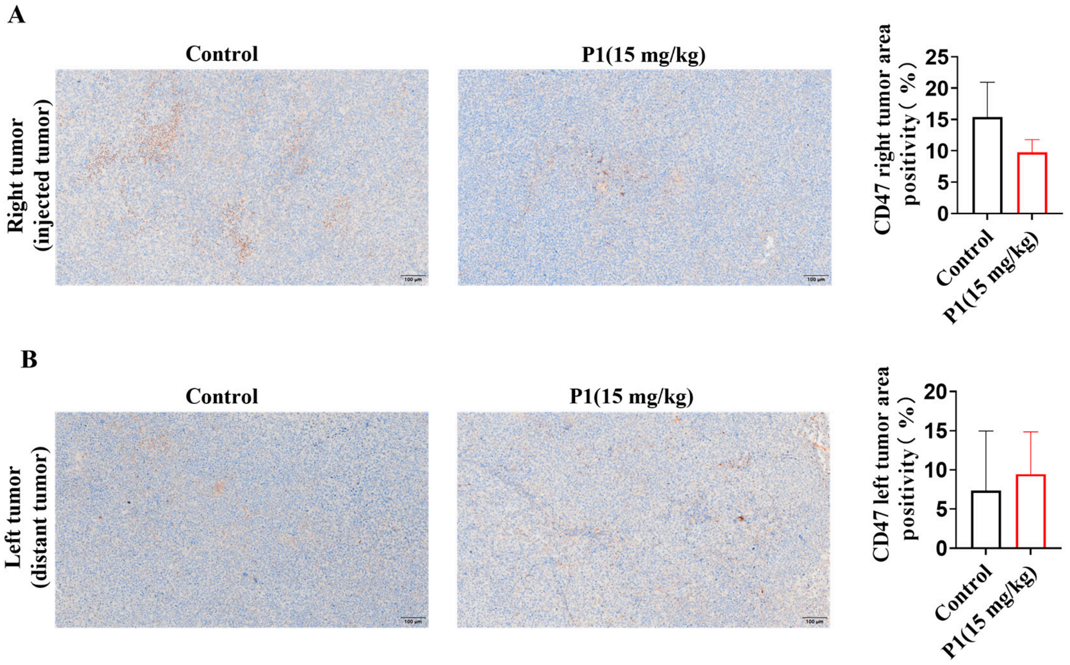

Figure S2. (**A**,**B**) IHC examination of CD47 in right tumor tissue (**A**) and in left tumor tissue (**B**). Scale bar, 100 µm (n=5).
